# 2-Arylbenzofurans as Selective Cholinesterase Inhibitors: Design, Synthesis, and Evaluation as Alzheimer’s Disease Agents

**DOI:** 10.3390/biom16010178

**Published:** 2026-01-22

**Authors:** Giovanna Lucia Delogu, Michela Begala, Manuel Novás, Maria João Matos, Franca Piras, Sonia Floris, Francesca Pintus, Michele Mancinelli, Benedetta Era, Antonella Fais

**Affiliations:** 1 Department of Live and Environmental Sciences, University of Cagliari, Cittadella Universitaria, SS 554, Km 4.5, 09042 Monserrato, Italy; michelabegala@unica.it (M.B.); s.floris@unica.it (S.F.); fpintus@unica.it (F.P.); era@unica.it (B.E.); fais@unica.it (A.F.); 2 Departamento de Química Orgánica, Facultad de Farmacia, Universidade de Santiago de Compostela, 15782 Santiago de Compostela, Spain; manuel.novas.gonzalez@rai.usc.es; 3 BioFarma Research Group, Center for Research in Molecular Medicine and Chronic Diseases (CiMUS), University of Santiago de Compostela, 15782 Santiago de Compostela, Spain; 4 Department of Biomedical Science, University of Cagliari, Cittadella Universitaria, SS 554, Km 4.5, 09042 Monserrato, Italy; fpiras@unica.it; 5 Department of Industrial Chemistry “Toso Montanari”, University of Bologna, Viale del Risorgimento, 4, 40136 Bologna, Italy; michele.mancinelli@unibo.it

**Keywords:** 2-arylbenzofurans, cholinesterase inhibitors, docking studies

## Abstract

New arylbenzofuran derivatives were designed, synthesized, and evaluated as potential inhibitors of acetylcholinesterase (AChE) and butyrylcholinesterase (BChE). Five hybrid compounds (**31**–**35**) feature a 2-phenylbenzofuran core linked via a heptyloxy spacer to an *N*-methylbenzylamine moiety, to enhance interactions within the active site of BChE. Biological evaluation revealed that brominated derivatives **34** and **35** showed the highest cholinesterases (ChE) inhibition compared to their chlorinated analogs, with compound **34** showing the highest activity for both AChE (IC_50_ = 27.7 μM) and BChE (IC_50_ = 0.7 μM). These compounds proved to be non-cytotoxic and demonstrated significant antioxidant activity in SH-SY5Y cells exposed to hydrogen peroxide (H_2_O_2_), highlighting their potential to mitigate oxidative stress: a key pathological factor in Alzheimer’s disease. Structural activity analysis suggests that bromine substitution at position 7 and the presence of a seven-carbon linker are critical for dual ChE inhibition and selectivity towards BChE. ADMET prediction indicates favorable pharmacokinetic properties, including drug-likeness and oral bioavailability. Overall, these findings highlight the potential of the 2-arylbenzofuran as a promising scaffold for multitarget-directed ligands in Alzheimer’s disease therapy.

## 1. Introduction

Multitarget-directed ligands (MTDLs) are an interesting approach in medicinal chemistry, especially in drug discovery programs related to multifactorial diseases, like Alzheimer’s disease (AD). MTDLs have several advantages over multiple one-target agents, as they can simultaneously inhibit multiple targets, potentially modifying or slowing down the disease progression, improving both therapeutic efficacy and safety. Additionally, administering MTDLs is easier because of their single-molecule administration, which is a significant benefit for AD patients presenting memory impairments [1,2].

**Figure 1:**
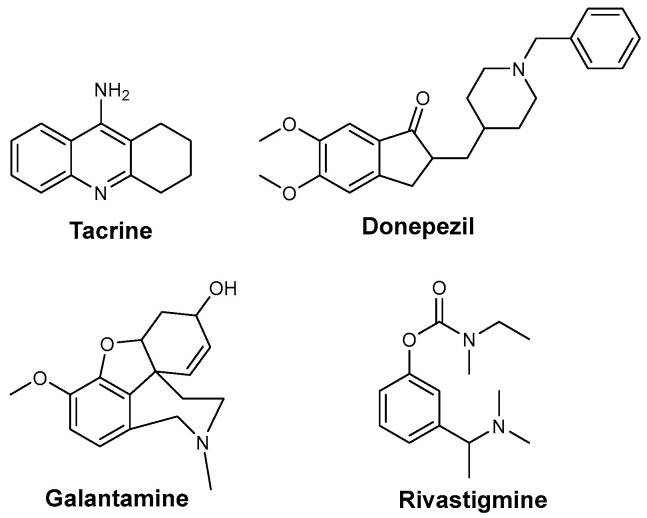
Chemical structures of four drugs approved by the FDA for AD. After some years being used, tacrine has been discontinued due to severe side effects.

In the brains of AD patients, the cholinergic system is most severely affected, with a significant depletion of acetylcholine (ACh) and other markers of cholinergic activity. This cholinergic hypothesis, proposed over 40 years ago, suggests that dysfunction of ACh-containing neurons in the brain plays a major role in the cognitive decline observed in aging and AD [3]. Therefore, one potential treatment approach involves a boost in the cholinergic levels in the brain by inhibiting acetylcholinesterase (AChE), an enzyme responsible for the decreasing levels of ACh. Four drugs (tacrine, donepezil, rivastigmine, and galantamine) have been approved by the FDA to help to mitigate AD symptoms (Figure 1). Although tacrine was initially approved by the FDA, it was discontinued due to side effects in 2013. Their mechanism of action involves inhibiting the active site of AChE, which leads to increased levels in the neurotransmitter in the synaptic cleft. This therapeutic approach provides only palliative effects and does not stop the progression of the disease. However, AChE inhibitors can still reduce cognitive and functional symptoms by enhancing cholinergic neurotransmission, thereby offering symptomatic relief. Additionally, these drugs have been associated with various side effects that tend to increase with higher doses.

An important role is also played by butyrylcholinesterase (BChE), the second member of the cholinesterase (ChE) family. In fact, BChE appears to be involved in the hydrolysis of ACh during the late stages of the disease, contributing to the breakdown of this important neurotransmitter [4].

The progressive accumulation of β-amyloid (Aβ) in AD has generally been considered fundamental to the development of this neurodegenerative disease. Cell toxicity associated with Aβ may explain the neuronal cell loss observed during the pathology. Therefore, preventing cellular neurodegeneration by inhibiting the formation of toxic forms of Aβ is a potential therapeutic approach that is being explored [5]. In fact, lecanemab, an Aβ directed monoclonal antibody, was approved by the FDA in 2023 [6]. The approval of lecanemab was controversial, as its safety–efficacy balance was highly debated. Even though it was able to reduce levels of Aβ, as shown in amyloid PET studies, its efficacy was not up to par. The effects on cognition were measured using the CDR-SB scale, demonstrating an absolute difference with placebo of +0.45 after 18 months of treatment [7]. For this scale, the minimal clinically important difference (MCID) has been established by experts at one to two points [8]. In addition, it is only approved by the FDA for patients who carry one or none of the apolipoprotein E ε4 (ApoE ε4) genes, because of the higher risk of amyloid-related imaging abnormalities (ARIA) in other patients. This has led scientists to partially discard the amyloid hypothesis for others, such as the tau or neuroinflammatory hypothesis [9]. Nevertheless, every fully penetrant mutation of AD increases the Aβ42/40 ratio and the protective APP A673T mutation reduces it [10]. This raises the question of whether the Aβ form that is being tackled (plaques or fibrils over oligomers) or the time when it is being tackled (late-stage AD) is what is preventing treatments from being ineffective.

**Figure 2:**
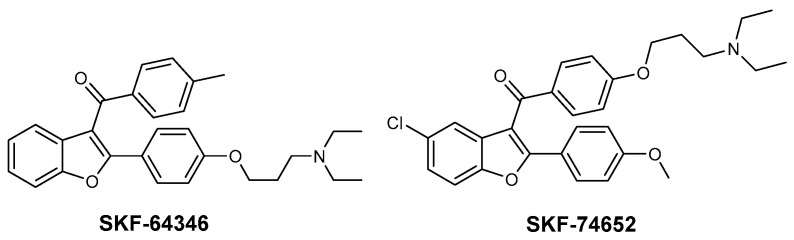
The 2,3-Disubstituted-benzofuran derivatives (SKF).

Howlett et al. described a series of benzofuran derivatives: specifically, 2,3-disubstituted (SKF), identified as inhibitors of Aβ fibril formation (Figure 2). These compounds proved to be effective in preventing the development of fibrillar Aβ, likely through a process that involves the benzofuran binding to the peptide [11].

**Figure 3:**
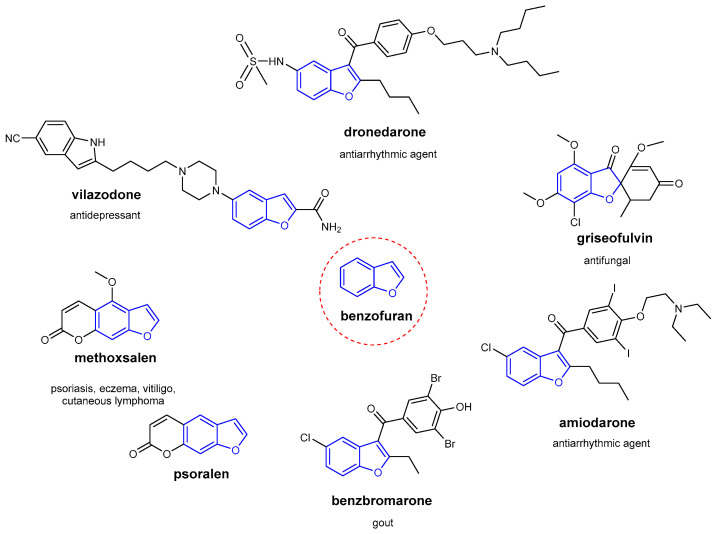
Some approved drugs containing benzofuran scaffolds (highlighted in blue).

Heterocyclic compounds, including benzofuran derivatives, play a crucial role in medicinal chemistry, due to their structural diversity and valuable physicochemical properties. Several approved drugs, either synthetic or naturally occurring compounds, contain the benzofuran scaffold, both mono- and fused-benzofuran rings, which is often combined with other heterocyclic systems [12]. Benzofuran derivatives (Figure 3) exhibit a broad spectrum of remarkable biological and pharmacological activities, including anti-tumor, antibacterial, antioxidant, anti-AD, anti-parasitic, anti-inflammatory, and bone anabolic properties. They are also promising in the development of multifunctional drugs, such as anti-proliferative agents for cancer treatment and compounds that inhibit amyloid aggregation in AD [13].

**Figure 4:**
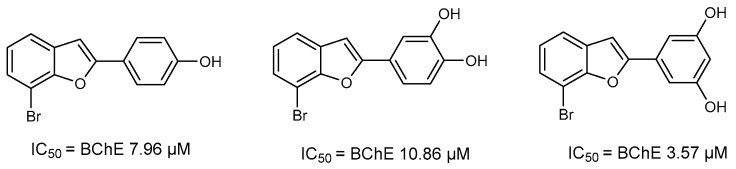
Most potent inhibitors from the studied series of 7-bromo-2-phenylbenzofurans.

Our research group has been working for many years on the design and synthesis of AChE and BChE inhibitors as potential drugs for AD. In some of our previous papers, we reported the design, synthesis, and biological evaluation of a series of hydroxylated 2-phenylbenzofuran derivatives [14,15,16]. The experimental results demonstrated that most benzofurans tested are selective BChE inhibitors, exhibiting different levels of effectiveness. The IC_50_ values for BChE inhibition revealed that compounds containing a bromine atom at position 7 of the benzofuran scaffold and at least one hydroxyl group in the *meta*-position of the 2-phenyl ring are the most potent inhibitors, with values ranging from 3.57 to 10.86 µM (Figure 4).

This work represents a step forward in the study of this scaffold, offering a comprehensive understanding of how the number and position of hydroxyl groups and halogen atoms may influence the properties of the 2-phenylbenzofuran core, as well as the interest in increasing a side chain at the hydroxyl group, making some new hybrid compounds.

**Figure 5:**
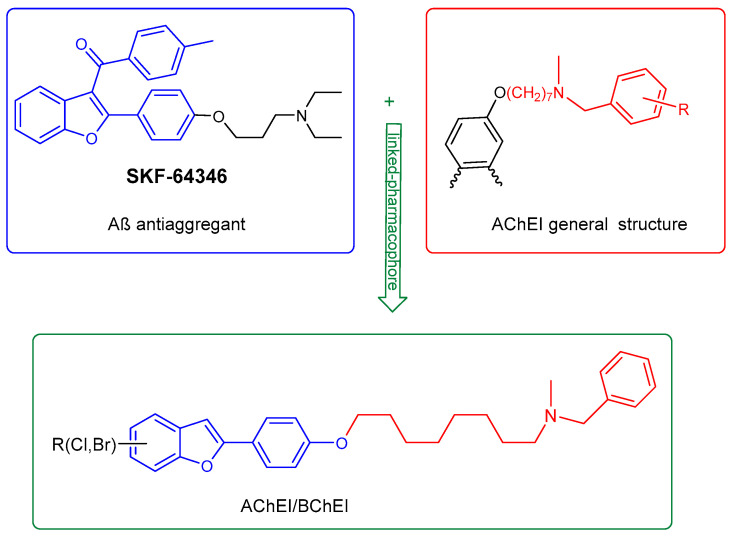
Rational design for the studied family of hybrid compounds.

To optimize and enhance the biological activities of the benzofuran scaffold, and to further explore the chemical space in interactions with the selected targets, this work presents the design, synthesis, molecular docking, and biological evaluation of eight new compounds (compounds **27**–**30** and **32**–**35**). Inspired by the work of Rampa and Piazzi, five of the new molecules (compounds **31** to **35**) contain a 2-phenylbenzofuran ring, connected via an appropriate heptyloxy spacer to an *N*-methylbenzylamine group (Figure 5) [17,18]. The *N*-methylbenzylamine group is a chemical structure found in certain molecules that are currently being investigated as potential treatments for AD, such as xanthostigmine, a potent AChE inhibitor [19,20]. On the other hand, the heptyloxy chain enables the molecule to reach both the deeper and more external regions of the enzyme’s active site [21].

The workflow for this project followed a medicinal chemistry point of view. First, the new chemical compounds were designed, synthesized, and fully characterized. Theoretical calculations have been performed to predict their potential as AChE and BChE ligands, as well as their pharmacokinetic properties. Then, cell viability has been screened to better understand their potential as future drug candidates. Finally, they have been studied for their AChE and BChE inhibitory activities, together with their antioxidant potential.

## 2. Materials and Methods

### 2.1. Chemistry

The starting materials, solvent, and reagents were obtained from commercial suppliers (Sigma-Aldrich-St. Luis, MO, USA) and were used without further purification. All reactions were performed under N_2_ atmosphere. Analytical thin-layer chromatography (TLC) was carried out on silica gel 60 F254 plates (0.25 mm), visualized by exposure to UV light (254 nm). Column chromatography purifications were performed using Aldrich silica gel on mesh size (60–120). Melting points were determined on a Stuart Scientific SMP 11 melting point apparatus and are uncorrected. Concentration and evaporation of the solvent after reaction or extraction were carried out on a rotary evaporator (IKA RV 10 Digital V Rotary Evaporator) operating at a reduced pressure. ^1^H NMR and ^13^C NMR spectra were recorded with a spectrometer (Bruker Avance III HD 600), operating at a field of 14.4 T (600 MHz for ^1^H and 150.8 MHz for ^13^C), using CDCl_3_ as a solvent. Chemical shifts are reported in ppm (δ), relative to TMS (tetramethylsilane), as an internal standard. The 150.8 MHz ^13^C spectra were acquired under proton decoupling conditions with a 36,000 Hz spectral width, 5.5 µs (60° tip angle) pulse width, 1 s acquisition time, and 4 s delay time. The long relaxation time was needed to observe some quaternary carbons. Coupling constants, J, are expressed in hertz (Hz). Spin multiplicities are given as s (singlet), d (doublet), dd (doublet of doublets), m (multiplet), and apparent triplet (app t). GC-MS low-resolution mass spectrometric experiments were carried out on a Saturn 2000 ion-trap coupled with a Varian 3800 gas chromatograph (Varian, Walnut Creek, CA, USA), operating under EI conditions (electron energy 70 eV, emission current 20 mA, ion-trap temperature 200 °C, manifold temperature 80 °C, automatic gain control (AGC) target 21,000), with the ion trap operating in scan mode (scan range from *m*/*z* 40–600 at a scan rate of 1 scan/s). Aliquots of 1 µL of solutions 1.0 × 10^−5^ M in dichloromethane (DCM) have been introduced into the gas chromatographer inlet. An Agilent J&W VF-5ms Low-bleed/MS GC capillary column (30 m, 0.25 mm i.d., 0.25 mm film thickness) (Agilent Technologies Inc., Wilmington, DE, USA) was used. The oven temperature was programmed from 100 °C (held for 2 min) to 325 °C, at 30 °C/min (held for 10 min). The temperature was then ramped up to 350 at 20 °C/min. The transfer line was maintained at 250 °C and the injector port (30:1 split) at 290 °C. HRMS-positive ESI-MS spectra were recorded with a high-resolution LTQ Orbitrap Elite™ mass spectrometer (Thermo Fisher Orbitrap Elite). The solutions were infused at a flow rate of 5.00 μL/min into the ESI source. Spectra were recorded in the range of *m*/*z* 100–1500 with a resolution of 240,000. The instrumental conditions were as follows: a spray voltage of 3500 V, a capillary temperature of 275 °C, sheath gas at 5–10 (arbitrary units), auxiliary gas at 3 (arbitrary units), sweep gas at 0 (arbitrary units), and a probe heater temperature of 50 °C.

### 2.2. Biological Activity

#### 2.2.1. Determination of Cholinesterase’s Inhibition

For the inhibition assays, AChE (EC 3.1.1.7) from *Electrophorus electricus* and BChE (EC 3.1.1.8) from equine serum were employed. Cholinesterase activity was assessed using Ellman’s colorimetric method [22], with minor adjustments to previously reported protocols [23]. The reaction mixture (final volume 200 µL) consisted of acetylthiocholine iodide (1.5 mM), 5,5′-dithiobis-(2-nitrobenzoic acid) (DTNB, 1.5 mM), and either the test compound at the desired concentration or DMSO as a control, which were all dissolved in 0.1 M phosphate buffer (pH 8.0). The enzyme was added immediately before measurement, and absorbance was recorded at 405 nm. The assay relied on AChE-mediated hydrolysis of acetylthiocholine, yielding acetate and thiocholine; thiocholine subsequently reacted with DTNB to form a yellow-colored anion.

For BChE inhibition, the same procedure was applied, substituting BChE as the enzyme and S-butyrylthiocholine chloride (BTCI) as the substrate. Half-maximal inhibitory concentrations (IC_50_), defined as the compound concentration producing 50% inhibition of enzymatic activity, were determined by fitting dose–response curves (see Supplemental Material). The reported IC_50_ values represent mean ± standard deviation from three independent experiments. Galantamine was used as a positive control under identical experimental conditions

#### 2.2.2. Cell Viability

The human neuroblastoma cell line SH-SY5Y was purchased from Professor Sogos (University of Cagliari, Italy) at the ICLC cell Bank (cat. # HTL95013). The cells were cultured in high glucose DMEM supplemented with 10% fetal bovine serum, 100 units/mL of penicillin, and 100 µg/mL of streptomycin (all from Gibco, Life sciences; Thermo Fisher Scientific, Waltham, MA, USA), and maintained at 37 °C in a humidified atmosphere of 5% CO_2_ and 95% air. Cell viability was assessed using the colorimetric 3-(4,5-dimethylthiazol-2-yl)-2,5-diphenyltetrazolium bromide (MTT) assay, as previously described, with minor modifications [15]. Briefly, 1.5 × 10^4^ cells/well were seeded in 96-well plates and incubated for 24 h with different concentrations (3–30 μM) of compounds. After incubation, 100 μL of MTT reagent (0.5 mg/mL in DMEM) was added, and cells were incubated for 3 h at 37 °C. The resulting violet formazan precipitate was dissolved in DMSO, allowing for quantification at 570 nm by spectrophotometry, using a microplate reader (VANTAstar_BMG LABTECH GmbH, Germany). Viability data were reported as a percentage of control for each compound.

### 2.3. Computational Methods

#### 2.3.1. Calculation of ADME Properties

SwissADME and ADMETlab 3.0 are web-based tools that are designed to predict the pharmacokinetic and toxicity profiles of small molecules. SwissADME focuses on evaluating drug-likeness, medicinal chemistry friendliness, and key properties such as absorption, distribution, metabolism, excretion (ADME), physicochemical characteristics, lipophilicity, water solubility, and potential bioavailability. ADMETlab 3.0 provides a comprehensive and accurate in silico assessment of ADMET properties, including potential toxicological risks, thereby supporting drug discovery and development processes [24,25].

#### 2.3.2. Molecular Docking

The three-dimensional protein structure of human BChE was obtained on ProteinDataBank (4TPK). Docking was performed on chain A of the enzyme, using the same coordinates as the ligand *N*-((1-(2,3-dihydro-1*H*-inden-2-yl)piperidin-3-yl)methyl)-*N*-(2-methoxyethyl)-2-naphthamide, which had been co-crystallized with the protein (7,12,11) Å. The size of the search space was established at (20,20,20) Å. The three-dimensional protein structure of human AChE was obtained on ProteinDataBank (4EY6). Docking was performed on chain A of the enzyme, using the same coordinates as the ligand (-)-galantamine that had been co-crystallized with the protein (−10,−43,30) Å. The size of the search space was established at (20,20,20) Å. Proteins were prepared using CHARMM. The missing side chains and hydrogen atoms were added, crystallographic ligands were removed, and protonation states were assigned at a physiological pH (7.4). All crystallographic water molecules were removed unless directly mediating ligand–active site interactions. Ligand structures (compounds **24**, **29**, and **34**) were generated from SMILES using OpenBabel, 3D-optimized using the MMFF94 force field, and protonated at pH 7.4. Gasteiger partial charges were assigned prior to docking. Molecular docking was carried out using AutoDock Vina v1.2.5 through the Vina Python API 3.8 (via SwissDock interface), establishing exhaustiveness at 8. Protein–ligand interactions were analyzed using PLIP v2025, identifying hydrogen bonds, π–π stacking, and hydrophobic interactions. Docking poses and interaction diagrams were visualized in PyMOL 3.10, and figures were exported at 300 DPI [26].

### 2.4. Statistical Analysis

Data are expressed as mean ± standard deviation (SD). One-way ANOVA and Tukey’s post hoc test were performed for group comparison, using GraphPad Prism software v. 8 (San Diego, CA, USA).

## 3. Results and Discussion

### 3.1. Chemistry

**Scheme 1:**
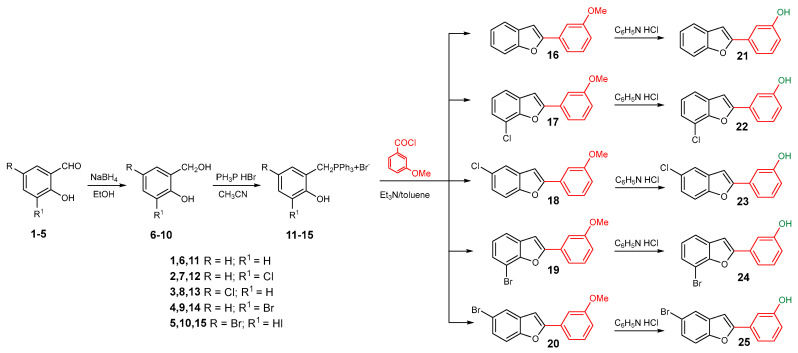
Synthesis of 2-hydroxyphenylbenzofurans (**21** to **25**).

**Scheme 2:**
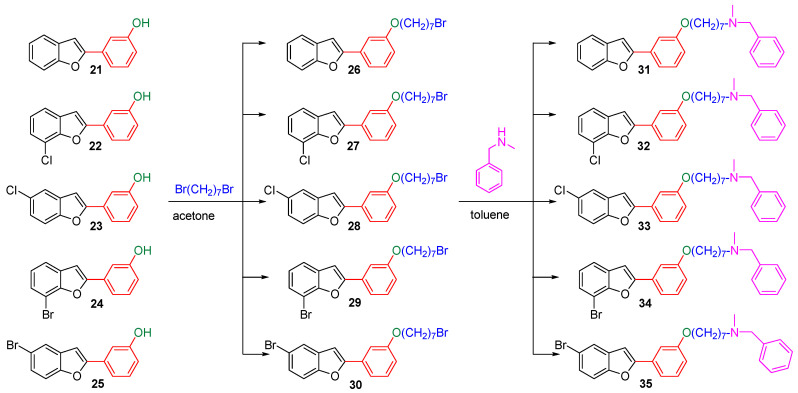
Synthesis of 2-hydroxyphenylbenzofurans ethers (**26** to **35**).

The synthesis of the studied compounds was carried out as illustrated in Scheme 1 and Scheme 2. The key step involves a modified intramolecular Wittig reaction, which efficiently yields the methoxylated benzofuran derivatives. Wittig reagents **11**–**15** were prepared by coupling 2-hydroxybenzyl alcohols **6**–**10** with triphenylphosphine hydrobromide in acetonitrile, at 82 °C. The resulting phosphonium salts were then reacted with 3-methoxybenzoyl chloride, in anhydrous toluene, in the presence of triethylamine, to afford the 2-(3-methoxybenzoyl)benzofurans **16**–**20**. Hydroxy derivatives 2-(3-hydroxyphenyl)benzofurans **21**–**25** were obtained from the corresponding methoxy compounds by heating a mixture of the methyl aryl ether and pyridine hydrochloride in a stopped round bottom flask under microwave irradiation (300 W) for 10–15 min, in a near quantitative yield (Scheme 1). The Wittig reagents and the methoxylated and hydroxylated benzofurans have previously been described by our research group [14,15,16,27,28].

The hydroxylated benzofuran derivatives were *O*-alkylated by treatment with 1,7-dibromoheptane to afford the corresponding halo-alkyloxy derivatives **26**–**30**. Subsequently, condensation with *N*-methylbenzylamine, under reflux in toluene, afforded derivatives **31**–**35** (Scheme 2).

The chemical structures of all the synthesized compounds were confirmed by different spectroscopic techniques, such as ^1^H NMR, ^13^C NMR, and mass spectrometry (information available in the Supporting Information).

### 3.2. Biological Activity

The inhibitory properties of the synthesized compounds toward ChE were evaluated using *Electrophorus electricus* AChE and equine serum BChE, which were selected for their availability and high degree of homology to the corresponding human enzymes. These enzyme sources are widely used for preliminary screening and represent suitable in vitro models. IC_50_ values were determined for compounds that exhibited at least 40% inhibitory activity at a concentration of 50 µM. The resulting inhibitory activities are summarized in Table 1.

Five halo-alkyloxy derivatives (**26**–**30**) and five benzofuran-*N*-methylbenzylamine hybrid derivatives (**31**–**35**) were synthesized and evaluated for their AChE and BChE inhibitory activities, together with the reference compound galantamine. As shown in Table 1, halo-alkyloxy derivatives do not show appreciable inhibitory activity. As for the benzofuran-*N*-methylbenzylamine hybrid derivatives, those substituted with a bromine atom at positions 5 and 7 of the benzofuran ring (**34** and **35**) exhibited higher activity compared to their chlorinated derivatives (**32** and **33**).

The most potent compound in the series against BChE was benzofuran **34**, with an IC_50_ value of 0.7 μM. Its positional isomer, benzofuran **35**, bearing a bromine atom at position 5, was five times less active (IC_50_ = 3.5 μM), although the difference was not statistically significant. Compound **32**, bearing a chlorine atom at position 7, exhibited an IC_50_ value of 10.4 μM, while compound **33** with chlorine at position 5 was the least active among the five hybrid compounds. Remarkably, compounds **31** to **35**, except compound **33**, demonstrated higher inhibitory activity against BChE compared to galantamine, the reference standard. The unsubstituted benzofuran **31** exhibited good inhibition of BChE, with an IC_50_ value of 2.1 μM, which is statistically equal to those of compounds **34** and **35** and comparable to the value reported by Rizzo et al. [29]. The presence of a bromine atom at position 5 or 7 of the benzofuran ring significantly increases the inhibitory activity compared to derivatives bearing chlorine at the same positions.

The new brominated compounds (**34** and **35**) demonstrated a statistically significant (*p* = 0.0015) improvement in BChE inhibitory activity compared to the parent compounds (**24** and **25**). Furthermore, all news compounds, except for compound **33**, showed improved inhibitory activity compared to the heptyloxyl derivatives (**26**–**30**).

Regarding the inhibitory activity of the compounds against AChE, the most active compounds were still **34** and **35**, with IC_50_ values of 27.7 μM and 32.2 μM, respectively. The unsubstituted benzofuran **31** exhibited an IC_50_ value of 43.2 μM, whereas the same compound reported by Rizzo et al. [29] was found to be inactive.

Interestingly, compounds **34** and **35** proved to be the best AChE and BChE inhibitors from the hybrid series. Heptyloxy derivatives **26**–**30** proved to be inactive against both enzymes, making the *N*-methylbenzylamine moiety a very interesting chemical feature to explore. Overall, the presence of the bromine atom at position 5 or 7, together with a seven-carbon spacer chain linking the 2-phenylbenzofuran core to the *N*-methylbenzylamine moiety, proved to be key structural features for achieving dual AChE/BChE activity.

One of the most relevant results for these derivatives is that, with the introduction of the *N*-methylbenzylamine chain, a greater selectivity for BChE compared to AChE was obtained, as is evident from Table 1. The IC_50_ ratio of AChE and BChE revealed that compound **34** has a higher selectivity than the other compounds. The best IC_50_ values against AChE and BChE were observed for compounds **34** and **35**. Keep this in mind; to better understand the role of bromine substitution and chain elongation, further studies were carried out on these two compounds. Overall, these results indicate that the combined introduction of a bromine substituent on the benzofuran core and a flexible aminoalkyl chain significantly enhances both the inhibitory activity and the selectivity toward BChE, representing a clear improvement over the previously reported hydroxylated 2-phenylbenzofuran derivatives [14,15,16].

### 3.3. Cell Viability and Cellular Antioxidant Activity

**Figure 6:**
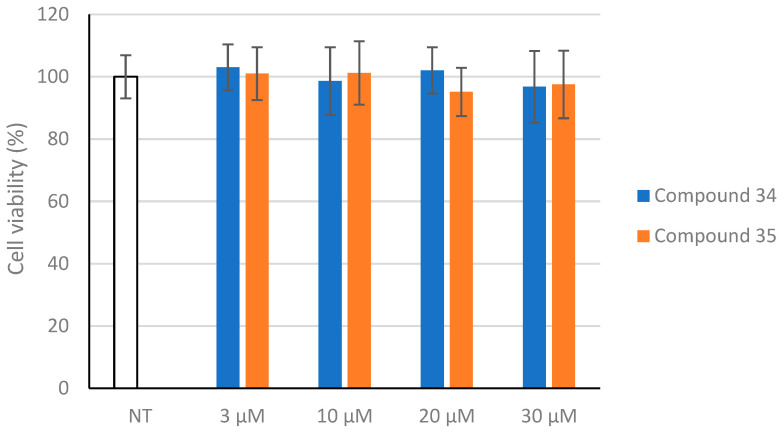
The effect of compounds **34** and **35** on SH-SY5Y cell viability. Data are expressed as a percentage of control NT (non-treated cells-white column). Data represent the mean (±standard deviation) of six independent experiments.

Based on promising results from earlier experiments, we conducted additional evaluations to assess the compounds’ cytotoxicity. We tested whether compounds **34** and **35** affect cell viability, using SH-SY5Y cells as a cellular model. Human neuroblastoma SH-SY5Y cells serve as an effective cellular model because they present with the AChE and BChE enzymes [30]. Cells were treated with different concentrations of the compounds for 24 h, then examined using the MTT assay. Our findings demonstrated that the compounds were noncytotoxic to SH-SY5Y cells at the concentration required to inhibit AChE and BChE activity. Statistical analysis was performed, and no significant differences were observed (ANOVA, *p* ≥ 0.999), Figure 6.

**Figure 7:**
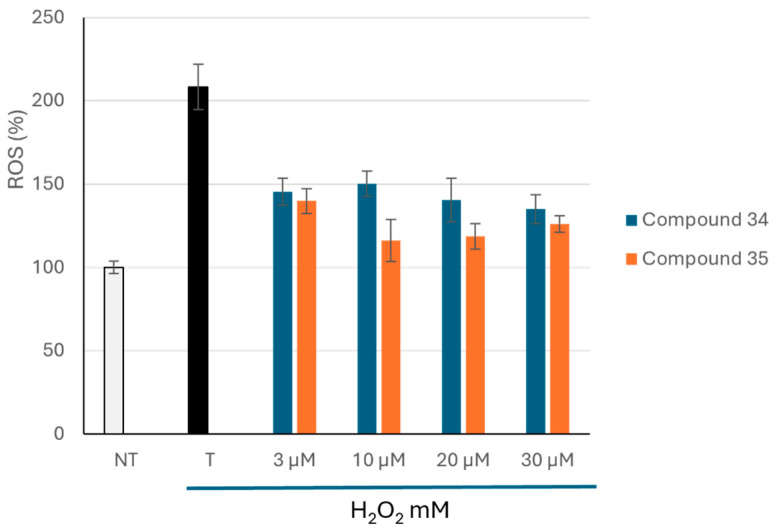
Inhibition of H_2_O_2_-induced ROS generation by compounds **34** and **35** on SH-SY5Y cells. NT, (non-treated cells-white column) and T, (treated cells-blak column) with H_2_O_2_ only. Data represent the mean (±standard deviation) of six independent experiments. All sample values are statistically different from cells treated with H_2_O_2_ alone (T), based on ANOVA (*p* < 0.0001) followed by Tukey’s post hoc test.

Given that oxidative stress plays an important role in many chronic diseases, including AD, this study evaluated the antioxidant activity of compounds **34** and **35** in SH-SY5Y cells exposed to hydrogen peroxide (H_2_O_2_). Reactive oxygen species (ROS) levels in cells were assessed before and after H_2_O_2_-induced oxidative stress and after treatment with compounds at different concentrations (3–30 μM). The results are shown in Figure 7.

### 3.4. Absorption, Distribution, Metabolism, and Excretion (ADMET) Studies

To be considered drug-like with favorable pharmacokinetic properties, compounds are generally expected to meet the criteria defined by Lipinski’s Rule of Five. The drug-likeness profiles of the potential AChE and BChE inhibitors, evaluated using the SwissADME program [24] and ADMETlab 3.0 program [25], are summarized in Table 2. Physicochemical properties such as molecular weight (MW), volume, number of rotatable bonds (RB), number of hydrogen bond acceptors (HBA) and donors (HBD), topological polar surface area (TPSA), lipophilicity, and aqueous solubility are critical parameters to be evaluated in a drug development program, at the early stages of compound design and optimization. Several filters have been used to calculate the physicochemical parameters and evaluate the drug-likeness of the synthesized compounds **26**–**35** [31,32,33,34,35].

For the compounds studied, MW varies between 387 and 506 g/mol, which falls within the optimum range for drug-likeness (150 ≤ MW g/mol ≤ 500). The numbers of HBA and HBD varied between 2 and 3, and 0, respectively, which are favorable values for the development of a drug candidate (HBA ≤ 10; HBD ≤ 5). In the structure of the benzofurans, the fraction of sp^3^-hybridized carbon atoms exceeds 0.25, imparting significant molecular flexibility and potentially improving target interaction. In addition, the TPSA values of the compounds range between 22.37 and 25.61 Å^2^, falling within the generally accepted optimal range of 20 < TPSA < 130 Å^2^ for drug-like molecules. Finally, the synthesized compounds exhibit logP values that are slightly above the optimal threshold of 4.15.

Table 2 also shows ESOL (estimated solubility in water) values and solubility categories for the synthesized compounds. ESOL estimates the aqueous solubility directly from the chemical structure [36]. Compounds **26**–**35** have values greater than −6; thus, they are classified as poorly soluble (−6 < ESOL < 0). The introduction of polar groups may help to overcome this limitation. Structural modifications to the benzofuran core may be necessary to address the critical issues highlighted by this software.

### 3.5. Molecular Docking Study

**Figure 8:**
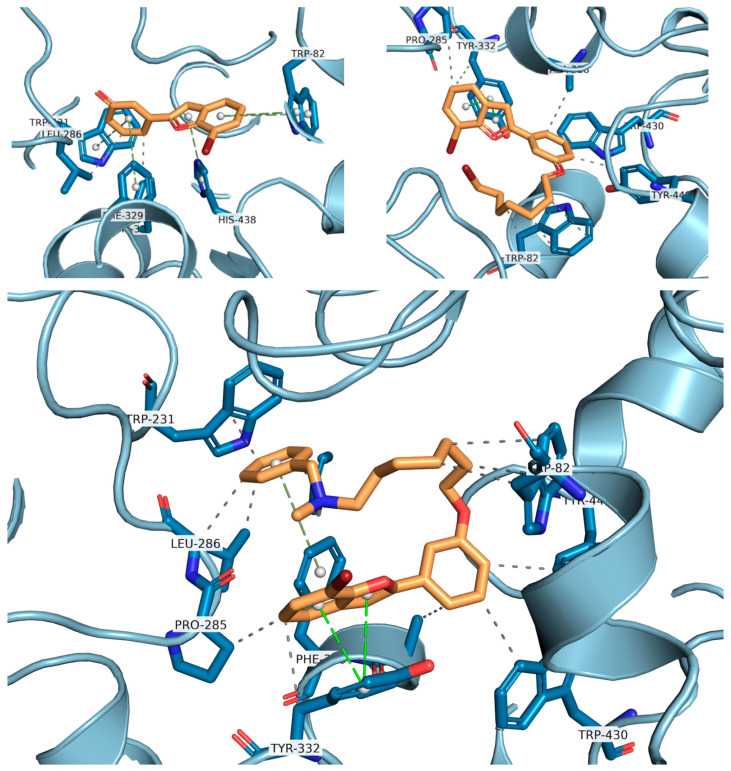
Predicted interactions of compounds **24** (**top-left**), **29** (**top-right**), and **34** (**bottom**) with BChE. Ligands are shown in orange and protein in blue (cartoon). Interacting residues are labeled. Hydrogen bonds are marked with a solid blue line, hydrophobic interactions with a dashed gray line, and π-stacking with a dashed green line (aromatic ring centers of the interaction are marked with a white sphere). PLIP data visualized using PyMOL.

Docking calculations have been performed to better understand the binding affinities of the different chemical constructs, and to help in the design of this series of compounds. The analysis of the data is aligned with the experimental observations for the best studied compounds: the derivatives **24** and **34**. Compound **24** (Figure 8) is able to interact with HIS438, which is part of the catalytic triad of the BChE, whereas this interaction is not present in the interactions predicted for compound **29** (Figure 8). This may explain its higher activity, as demonstrated by the lower IC_50_ value against this enzyme.

Compound **34**, on the other hand, establishes interactions with TRP82 and PHE329, part of the anionic site, the latter being a π-stacking interaction. Furthermore, it establishes a hydrophobic interaction with LEU286, which is part of the acyl pocket of the enzyme. These interactions may explain the lowest IC_50_ found for this hybrid molecule.

**Figure 9:**
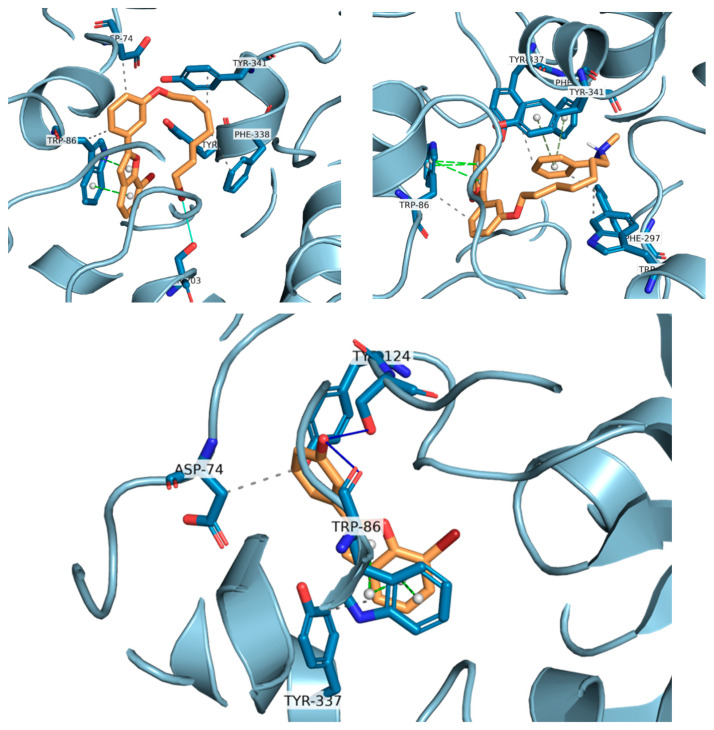
Predicted interactions of compounds **24** (**bottom**), **29** (**top-left**), and **34** (**top-right**) with AChE. Ligands are shown in orange and protein in blue (cartoon). Interacting residues are labeled. Hydrogen bonds are marked with a solid blue line, hydrophobic interactions with a dashed gray line, π-stacking with a dashed green line (aromatic ring centers of the interaction are marked with a white sphere). PLIP data visualized using PyMOL.

For compound **24**, the activity towards AChE can be explained by the formation of two hydrogen bonds, which are stronger interactions than the ones formed for compound **29**. These hydrogen bonds are formed due to the hydroxyl group, which is absent in molecule **29,** being able to interact with TRP86 and SER125A (Figure 9). Comparing compounds **29** and **34**, the latter presents a longer chain at the same position, which is able to establish several different hydrophobic interactions with multiple amino acids at the binding pocket (TYR337, TRP286, and PHE297) and π-stacking interactions due to the benzyl group with PHE338 and TYR341.

Calculated binding energies for compounds **24**, **29,** and **34** in AChE and BChE binding pockets can be found in the Supplementary Information (Tables S1–S6). Moreover, a comparison of the best predicted pose of compounds **24**, **29,** and **34** in the enzyme pockets can also be consulted in the Supplementary Information (Figure S1).

## 4. Conclusions

In this study, new 2-arylbenzofuran derivatives were synthesized and evaluated for their inhibitory activity against both ChE enzymes (AChE and BChE), using galantamine as a reference compound. Among the tested compounds, the brominated derivatives **34** and **35** showed superior inhibitory activity compared to their chlorine-substituted analogs. Notably, the new brominated compounds showed a statistically significant enhancement in BChE inhibitory activity compared to the original compounds. The unsubstituted benzofuran **31** also demonstrated good BChE inhibition, according to the activity that was previously reported in the literature. In contrast, chlorinated derivatives, particularly compound **33**, displayed the lowest ChE activity. Thus, the presence of a bromine atom and a seven-carbon linker that connects the benzofuran core to the *N*-methylbenzylamine side chain seem to be crucial structural features for achieving dual ChE inhibition. This structure also increases the inhibitory potency against BChE. The best compounds do not show cytotoxicity at concentrations that are equal to their IC_50_ value, and they show antioxidant activity in human neuroblastoma cells. A good correlation with the experimental data was observed from the molecular docking results. Compound **34** exhibits strong binding to BChE, accounting for its low IC_50_, while compound **29** lacks critical interactions, resulting in lower activity. These findings provide valuable insights for the future design of multitarget agents inspired by this scaffold for the treatment of AD. The ADMET profiling of the synthesized benzofurans highlights promising pharmacokinetic profiles for different compounds, particularly in terms of drug-likeness and oral bioavailability. Overall, these combined experimental and computational findings underscore the potential of this benzofuran scaffold as a promising basis for the development of future multitarget agents for Alzheimer’s disease therapy.

## Figures and Tables

**Table 1:** IC_50_ values of the 2-phenylbenzofuran derivatives **21**–**35** against AChE and BChE.

Compounds	IC_50_ AChE (µM)	IC_50_ BChE (µM)	Compounds	IC_50_ AChE (µM)	IC_50_ BChE (µM)	Compounds	IC_50_ AChE (µM) *	IC_50_ BChE (µM) *	Ratio (AChE/ BChE)
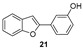	>50	>50	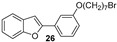	>50	>50	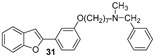	43.2 ± 0.9 ^a^	2.1 ± 0.1 ^a^	20.6
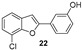	28.21 ± 1.26 [15]	13.42 ± 2.57 [15,16]	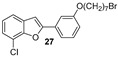	>50	>50	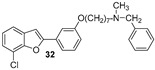	>50	10.4 ± 2.1 ^b^	-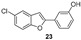	>50	10.5 ± 0.4 [14]	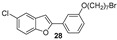	>50	>50	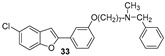	>50	>50	-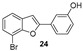	23.96 ± 2.77 [15]	7.96 ± 0.28 [15]	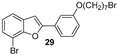	>50	>50	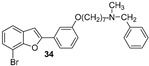	27.7 ± 1.48 ^b^	0.7 ± 0.01 ^a^	39.6
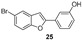	>100 [14]	10.6 ± 1.6 [14]	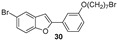	>50	>50	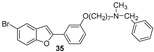	32.2 ± 1.8 ^b^	3.5 ± 0.6 ^a^	9.2
Galantamine	0.9 ± 0.02 [16]	28.3 ± 2.1 [16]							

IC_50_ values represent the mean ± standard deviation for three independent assays. * Different letters in each column, ^a^/^b^, indicate statistically significant differences between compounds (*p* < 0.0293 for AChE and *p* < 0.0007 for BChE, respectively, based on ANOVA followed by Tukey’s post hoc test.

**Table 2:** Physicochemical and pharmacokinetic properties of benzofurans **26–35**.

	Compounds	26	27	28	29	30	31	32	33	34	35
Physicochemical Properties	MW (g/mol)	387.31	421.76	421.76	466.21	466.21	427.58	462.02	462.02	506.47	506.47
	HBA	2	2	2	2	2	3	3	3	3	3
	HBD	0	0	0	0	0	0	0	0	0	0
	TPSA (Å^2^)	22.37	22.37	22.37	22.37	22.37	25.61	25.61	25.61	25.61	25.61
Lipophilicity	iLogP	4.59	4.79	4.87	4.89	4.90	5.39	5.62	5.64	5.73	5.74
Water Solubility	Log S (ESOL)	−6.32	−6.92	−6.92	−7.23	−7.23	−6.88	−7.48	−7.48	−7.79	−7.79
	Solubility	PWS	PWS	PWS	PWS	PWS	PWS	PWS	PWS	PWS	PWS
Pharmacokinetics	GI absorption	low	low	low	low	low	low	low	low	low	low
	BBB permeant	no	no	no	no	no	no	no	no	no	no
	Log Kp (cm/s)	−3.91	−3.68	−3.68	−3.90	−3.90	−3.61	−3.37	−3.37	−3.60	−3.60
Drug-Likeness	Lipinsky violations	1	1	1	1	1	1	1	1	2	2
	Ghose violations	1	1	1	1	1	2	2	2	3	3
	Veber violations	0	0	0	0	0	1	1	1	1	1
	Egan violations	1	1	1	1	1	1	1	1	1	1
	Muegge violations	1	1	1	1	1	1	1	1	1	1
	Bioavailability Score (F)	0.55	0.55	0.55	0.55	0.55	0.55	0.55	0.55	0.17	0.17

MW: molecular weight, TPSA: topological polar surface area, HBD: hydrogen bonds donor, HBA: hydrogen bond acceptor, iLogP values: indicator of lipophilicity, ESOL: aqueous solubility parameter, PWS: poorly water soluble; Log Kp: skin permeation, and F: bioavailability score.

## Data Availability

The data presented in this study are available upon request from the corresponding authors. The data are not publicly available, due to privacy.

## Supplementary Materials

The following supporting information can be downloaded at: https://www.mdpi.com/article/10.3390/biom16010178/s1. Procedure for the preparation compounds **26**–**35**, NMR, and Massa Spectra, Docking energies: Tables S1–S6, Figure S1: superposition compounds **24**, **29,** and **34**, [14,15,16,27,28,29].
