# Supplementary material for: 2-Arylbenzofurans as Selective Cholinesterase Inhibitors: Design, Synthesis, and Evaluation as Alzheimer’s Disease Agents
Source: Biomolecules. 2026 Jan 22;16(1):178. doi: 10.3390/biom16010178 (PMC12839115; doi:10.3390/biom16010178)
Supplement: Supplementary file 1 [file biomolecules-16-00178-s001.zip › biomolecules-4065259-supplementary.pdf]

Supplementary Material

Table of Contents

1. **General Experimental Information** S2

1.1 General procedure for the preparation of 2-[3-(7-bromoheptyloxy)-phenyl]-benzofurans **26-30** S3

1.2 2-[3-(7-Bromoheptyloxy)-phenyl]-benzofuran **26** S3

1.3 7-Chloro-2-[3-(7-bromoheptyloxy)-phenyl]-benzofuran **27** S4

1.4 5-Chloro-2-[3-(7-bromoheptyloxy)-phenyl]-benzofuran **28** S4

1.5 7-Bromo-2-[3-(7-bromoheptyloxy)-phenyl]-benzofuran **29** S5

1.6 5-Bromo-2-[3-(7-bromoheptyloxy)-phenyl]-benzofuran **30** S5

1.7 General procedure for the preparation of 7-[3-(benzofuran-2-yl)-phenoxy]-*N*-benzyl-*N*-methylheptanamines **31-35** S6

1.8 7-[3-(Benzofuran-2-yl)-phenoxy]-*N*-benzyl-*N*-methylheptanamine **31** S6

1.9 7-[3-(7-Chlorobenzofuran-2-yl)-phenoxy]-*N*-benzyl-*N*-methylheptanamine **32** S7

1.10 7-[3-(5-Chlorobenzofuran-2-yl)-phenoxy]-*N*-benzyl-*N*-methylheptanamine **33** S8

1.11 7-[3-(7-Bromobenzofuran-2-yl)-phenoxy]-*N*-benzyl-*N*-methylheptanamine **34** S8

1.12 7-[3-(5-Bromobenzofuran-2-yl)-phenoxy]-*N*-benzyl-*N*-methylheptanamine **35** S9

2 **NMR and Massa Spectra** S11

3 **Molecular Docking** S31

4 **References** S38

1. General Experimental Information

Starting materials, solvent and reagents were obtained from commercial suppliers (Sigma-Aldrich) and were used without further purification. All reactions were performed under N_2 atmosphere. Analytical thin layer chromatography (TLC) was carried out on silica gel 60 F254 plates (0.25 mm), visualized by exposure to UV light (254 nm). Column chromatography purifications were performed using. Aldrich silica gel (60-120) mesh size. Melting points were determined on a Stuart Scientific SMP 11 melting point apparatus and are uncorrected. Concentration and evaporation of the solvent after reaction or extraction were carried out on a rotary evaporator (Büchi Rotavapor) operating at reduced pressure. ^1H NMR and ^{13}C NMR spectra were recorded with a spectrometer (Varian INOVA) operating at a field of 14.4 T (600 MHz for ^1H, 150.8 MHz for ^{13}C) and using $CDCl_3$ as solvent. Chemical shifts are reported in ppm (δ) relative to TMS (tetramethylsilane) as an internal standard. The 150.8 MHz ^{13}C spectra were acquired under proton decoupling conditions with a 36000 Hz spectral width, 5.5 μs (60° tip angle) pulse width, 1 s acquisition time and 4 s delay time. The long relaxation time was needed to observe some quaternary carbons. Coupling constants *J* are expressed in hertz (Hz). Spin multiplicities are given as s (singlet), d (doublet), dd (doublet of doublets), m (multiplet) and apparent triplet (app t). GC-MS: low resolution mass spectrometric experiments were carried out on a Saturn 2000 ion-trap coupled with a Varian 3800 gas chromatograph (Varian, Walnut Creek, CA) operating under EI conditions (electron energy 70 eV, emission current 20 mA, ion-trap temperature 200 °C, manifold temperature 80 °C, automatic gain control (AGC) target 21.000) with the ion trap operating in scan mode (scan range from m/z 40-600 at a scan rate of 1 scan/s). Aliquots of 1 μL of solutions 1.0 x 10^{-5} M in dichloromethane (DCM) have been introduced into the gas chromatographer inlet. An Agilent J&W VF-5ms Low-bleed/MS GC capillary column (30 m, 0.25 mm i.d., 0.25 mm film thickness) (Agilent Technologies Inc., Wilmington, DE, USA), was used. The oven temperature was programmed from 100°C (held for 2 min) to 325 °C at 30 °C/min (held for 10 min). The temperature was then ramped to 350 at 20 °C/min. The transfer line was maintained at 250 °C and the injector port (30:1 split) at 290 °C. HRMS: positive ESI-MS spectra were recorded with

a high-resolution LTQ Orbitrap Elite™ mass spectrometer (Thermo Fisher Scientific). The solutions were infused at a flow rate of 5.00 μL/min into the ESI source. Spectra were recorded in the range of m/z 100-1500 with a resolution of 240000. The instrumental conditions were as follows. Spray voltage 3500 V, capillary temperature 275 °C, sheath gas 5-10 (arbitrary units), auxiliary gas 3 (arbitrary units), sweep gas 0 (arbitrary units), probe heater temperature 50 °C.

1.1 ***General procedure for the preparation 2-[3-(7-bromoheptyloxy)-phenyl]-benzofuran 26-30*** [1]

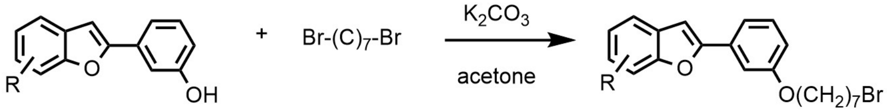

A stirred mixture of 2-(3-hydroxyphenyl)-benzofuran (1.0 equiv.) [2], 1,7-dibromoheptane (2.0 equiv.) and K_2CO_3 (2.0 equiv.) was refluxed in acetone dry for 20 h. The suspension was filtered while hot and the solvent was removed under reduced pressure. The crude product was purified by chromatography on silica gel using petroleum ether/dichloromethane mixture as the eluent to give the pure compound **26-30**.

1.2 ***2-[3-(7-Bromoheptyloxy)-phenyl]-benzofuran (26)*** [1]

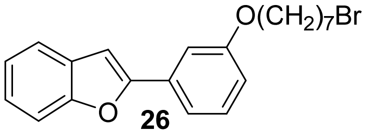

26

According to the procedure, using 2-(3-hydroxyphenyl)-benzofuran **21** (0.23 g, 0.11 mmol) and 1,7-dibromoheptane (0.38 mL, 0.22 mmol), K_2CO_3 (0.30 g, 0.22 mmol) and acetone (20 mL). Purification by column chromatography on silica gel in petroleum ether/dichloromethane 3:2. **26** was obtained as a white solid (0.03 g, 81%); mp: 145-146 °C; ^{1}H NMR (600 MHz, $CDCl_3$): δ 7.59 (d, *J* = 7.6 Hz, 1H), 7.53 (d, *J* = 8.1 Hz, 1H), 7.45 (d, *J* = 7.7 Hz, 1H), 7.42 (s, 1H), 7.35 (t, *J* = 7.9 Hz, 1H), 7.29 (dd, *J* = 11.3, 4.0 Hz, 1H), 7.24 (t, *J* = 7.4 Hz, 1H), 7.02 (s, 1H), 6.89 (dt, *J* = 10.6, 5.1 Hz, 1H), 4.05

(t, J = 6.4 Hz, 2H), 3.46-3.41 (m, 2H), 1.93-1.80 (m, 4H), 1.57-1.47 (m, 4H), 1.46-1.40 (m, 2H) ppm; ^{13}C NMR (151 MHz, $CDCl_3$): δ = 159.49, 155.83, 154.87, 131.75, 129.85, 129.21, 124.32, 122.96, 120.94, 117.42, 114.94, 111.18, 110.87, 101.59, 67.98, 33.93, 32.75, 29.22, 28.56, 28.12, 25.95 ppm; HRMS (ESI): calcd. for $C_{21}H_{23}BrO_2$ $[M]^+$ = 386.0881; found = 386.0869.

1.3 **7-Chloro-2-[3-(7-bromoeptyloxy)-phenyl]-benzofuran (27)**

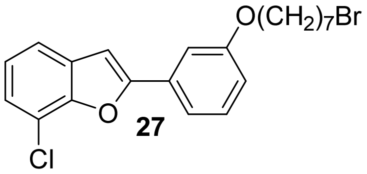

According to the procedure, using 7-chloro-2-(3-hydroxyphenyl)-benzofuran **22** (0.07 g, 0.33 mmol) and 1,7-dibromoheptane (0.10 mL, 0.66 mmol), K_2CO_3 (0.08 g, 0.66 mmol) and acetone (25 mL). Purification by column chromatography on silica gel in petroleum ether/dichloromethane 3:2. **27** was obtained as a yellowish oil (0.10 g, 77%); ^{1}H NMR (600 MHz, $CDCl_3$): δ = 7.47 (dd, J = 7.7, 1.0 Hz, 2H), 7.45-7.42 (m, 1H), 7.36 (t, J = 7.9 Hz, 1H), 7.28 (dd, J = 7.8, 0.9 Hz, 1H), 7.16 (t, J = 7.8 Hz, 1H), 7.04 (s, 1H), 6.92 (dd, J = 8.2, 2.5 Hz, 1H), 4.08-4.02 (m, 2H), 3.45-3.40 (m, 2H), 1.92-1.87 (m, 2H), 1.86-1.82 (m, 2H), 1.53-1.46 (m, 4H), 1.45-1.42 (m, 2H) ppm; ^{13}C NMR (151 MHz, $CDCl_3$): δ = 158.45, 155.68, 149.58, 130.08, 129.78, 128.89, 123.42, 122.81, 118.37, 116.58, 115.63, 114.12, 110.22, 100.96, 66.98, 32.91, 31.70, 28.17, 27.53, 27.08, 24.92 ppm; MS (EI, 70eV) *m/z* (%):
420 (73) [$M^{+\cdot}$ (^{79}Br, ^{35}Cl)], 422 (100) [M+2 (^{79}Br, ^{37}Cl) and (^{81}Br, ^{35}Cl)], 424 (25) [M+4 (^{81}Br, ^{37}Cl)], 244 (73) $[M\text{-}C_7H_{13}Br]^{+\cdot}$.

1.4 **5-Chloro-2-[3-(7-bromoeptyloxy)-phenyl]-benzofuran (28)**

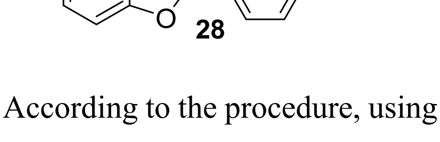

According to the procedure, using 7-chloro-2-(3-hydroxyphenyl)-benzofuran **23** (0.15 g, 0.60 mmol) and 1,7-dibromoheptane (0.20 mL, 1.20 mmol), K_2CO_3 (0.09 g, 1.20 mmol) and acetone (50 mL).

Purification by column chromatography on silica gel in petroleum ether/dichloromethane 7:3. **28** was obtained as a white solid (0.20 g, 83%); mp: 46-49 °C; ^{1}H NMR (600 MHz, $CDCl_3$): δ = 7.54 (d, *J* = 2.1 Hz, 1H), 7.43 (dd, *J* = 8.2, 5.1 Hz, 2H), 7.40-7.32 (m, 2H), 7.23 (dd, *J* = 8.7, 2.1 Hz, 1H), 6.96 (d, *J* = 10.5 Hz, 1H), 6.93-6.90 (m, 1H), 4.04 (t, *J* = 6.4 Hz, 2H), 3.42 (t, *J* = 6.8 Hz, 2H), 1.89 (m, 2H), 1.83 (m, 2H), 1.50 (m, 4H), 1.46-1.38 (m, 2H) ppm; ^{13}C NMR (151 MHz, $CDCl_3$): δ = 159.50, 157.32, 153.23, 131.21, 130.56, 129.93, 128.51, 124.44, 120.44, 117.51, 115.35, 112.12, 111.00, 101.06, 68.01, 33.91, 32.73, 29.19, 28.54, 28.10, 25.94 ppm; MS (EI, 70eV) *m/z* (%): 420 (69) [$M^{+\cdot}$ (^{79}Br, ^{35}Cl)], 422 (100) [M+2 (^{79}Br, ^{37}Cl) and (^{81}Br, ^{35}Cl)], 424 (28) [M+4 (^{81}Br, ^{37}Cl)], 244 (78) [M-C_7H_{13}Br]$^{+\cdot}$.

1.5 **7-Bromo-2-[3-(7-bromoeptyloxy)-phenyl]-benzofuran (29)**

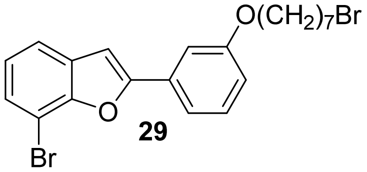

According to the procedure, using 7-bromo-2-(3-hydroxyphenyl)-benzofuran **24** (0.37 g, 1.3 mmol) and 1,7-dibromoheptane (0.44 mL, 2.6 mmol), K_2CO_3 (0.36 g, 2.6 mmol) and acetone (80 mL). Purification by column chromatography on silica gel in petroleum ether/dichloromethane 7:3. **29** was obtained as a white solid (0.50 g, 82%); mp: 28-30 °C; ^{1}H NMR (600 MHz, $CDCl_3$): δ 7.38 (d, *J* = 7.7 Hz, 1H), 7.35 (d, *J* = 7.7 Hz, 1H), 7.32 (dt, *J* = 3.6, 1.7 Hz, 2H), 7.24 (t, *J* = 7.9 Hz, 1H), 6.99 (t, *J* = 7.7 Hz, 1H), 6.93 (s, 1H), 6.80 (dd, *J* = 8.1, 2.3 Hz, 1H), 3.91 (t, *J* = 6.4 Hz, 2H), 3.31 (t, *J* = 6.8 Hz, 2H), 1.81-1.74 (m, 2H), 1.74-1.66 (m, 2H), 1.44-1.34 (m, 4H), 1.33-1.27 (m, 2H) ppm; ^{13}C NMR (151 MHz, $CDCl_3$): δ = 159.47, 156.58, 151.95, 131.07, 130.48, 129.93, 127.31, 124.26, 120.07, 117.61, 115.08, 111.31, 103.96, 102.14, 68.01, 34.00, 32.77, 29.76, 29.23, 28.59, 28.14, 25.98 ppm; MS (EI, 70eV) *m/z* (%): 464 (54) [$M^{+\cdot}$ (^{79}Br)], 466 (98) [M+2 (^{79}Br, ^{81}Br)], 468 (45) [M+4 (2 x ^{81}Br)], 384 (46) [M-HBr]$^{+\cdot}$, 288 (100) [M-C_7H_{13}Br]$^{+\cdot}$.

1.6 **5-Bromo-2-[3-(7-bromoeptyloxy)-phenyl]-benzofuran (30)**

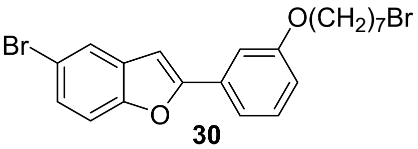

30

According to the procedure, using 5-bromo-2-(3-hydroxyphenyl)-benzofuran **25** (0.27 g, 0.93 mmol) and 1,7-dibromoheptane (0.32 mL, 1.86 mmol), K_2CO_3 (0.26 g, 1.86 mmol) and acetone (60 mL). Purification by column chromatography on silica gel in petroleum ether/dichloromethane 7:3. **30** was obtained as a white solid (0.36 g, 85%); mp: 40-42 °C; ^{1}H NMR (600 MHz, $CDCl_3$): δ = 7.69 (dd, *J* = 1.8, 0.7 Hz, 1H), 7.42 (dt, *J* = 7.8, 1.2 Hz, 1H), 7.40-7.32 (m, 4H), 6.94 (d, *J* = 0.8 Hz, 1H), 6.91 (ddd, *J* = 8.2, 2.6, 1.0 Hz, 1H), 4.04 (t, *J* = 6.4 Hz, 2H), 3.43 (t, *J* = 6.8 Hz, 2H), 1.92-1.86 (m, 2H), 1.86-1.80 (m, 2H), 1.51 (m, 4H), 1.45-1.39 (m, 2H) ppm; ^{13}C NMR (151 MHz, $CDCl_3$): δ 159.50, 157.15, 153.58, 131.20, 131.14, 129.94, 127.14, 123.50, 117.52, 116.01, 115.36, 112.62, 111.00, 100.91, 68.00, 33.95, 32.74, 29.20, 28.55, 28.11, 25.95 ppm; MS (EI, 70eV) *m/z* (%): 464 (48) [$M^{+\cdot}$ (^{79}Br)], 466 (96) [M+2 (^{79}Br, ^{81}Br)], 468 (45) [M+4 (2 x ^{81}Br)], 384 (46) $[M-HBr]^{+\cdot}$, 288 (95) $[M-C_7H_{13}Br]^{+\cdot}$.

1.7 **General procedure for the preparation of 7-[3-(benzofuran-2-yl)-phenoxy]-*N*-benzyl-*N*-methylheptanamines 31-35** [1]

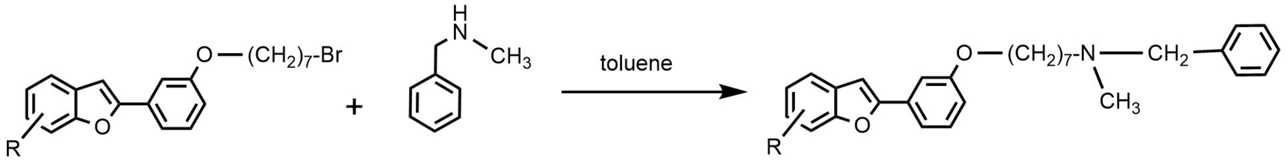

A stirred solution of **25-30** (1.0 equiv.) and *N*-methylbenzylamine (2.0 equiv.) in toluene was refluxed in the presence of a catalytic amount of NaI for 20 h. The mixture was washed with water (3 x 25 mL) and the organic layer was dried over Na_2SO_4. The solvent was removed under reduced pressure and the residue was purified by flash chromatography on silica gel using diethyl ether/acetone mixture as the eluent to give the pure compound **31-35**.

1.8 **7-[3-(Benzofuran-2-yl)-phenoxy]-*N*-benzyl-*N*-methylheptanamine (31)** [1]

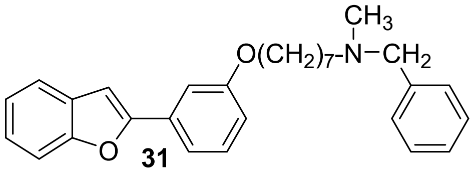
31

Using the previous procedure and starting from benzofuran **26** (0.50 g, 1.3 mmol), *N*-methylbenzylamine e (0.33 mL, 2.6 mmol) and NaI (0.19 g, 1.3 mmol) in toluene (100 mL). Purification by column chromatography on silica gel in diethyl ether/acetone 9.8:0.2. 31. **31** was obtained as yellowish oil: (0.30 g, 55% yield); ^{1}H NMR (600 MHz, $CDCl_3$): δ = 7.51 (dd, *J* = 7.7, 1.1 Hz, 1H), 7.47 (dt, *J* = 7.7, 1.3 Hz, 1H), 7.45-7.41 (m, 2H), 7.36 (t, *J* = 7.9 Hz, 1H), 7.33-7.28 (m, 4H), 7.26 (m, 2H), 7.10 (t, *J* = 7.8 Hz, 1H), 7.06 (s, 1H), 6.92 (ddd, *J* = 8.2, 2.5, 0.9 Hz, 1H), 4.04 (t, *J* = 6.5 Hz, 2H), 3.49 (s, 2H), 2.38 (dd, *J* = 8.4, 6.5 Hz, 2H), 2.19 (s, 3H), 1.86-1.80 (m, 2H), 1.58-1.47 (m, 4H), 1.42-1.32 (m, 4H) ppm; ^{13}C NMR (151 MHz, $CDCl_3$): δ = 159.63, 156.74, 152.09, 139.25, 131.20, 130.60, 130.03, 129.24, 128.32, 127.41, 127.04, 124.34, 120.15, 117.70, 115.24, 111.46, 104.08, 102.22, 68.24, 62.45, 57.60, 42.37, 29.47, 29.40, 27.50, 27.43, 26.20 ppm; HRMS (ESI): calcd. for $C_{29}H_{34}NO_2$ $[M+H]^+$ = 428.2589; found 428.2601.

1.9 **7-[3-(7-Chlorobenzofuran-2-yl)-phenoxy]-*N*-benzyl-*N*-methylheptanamine (32)**

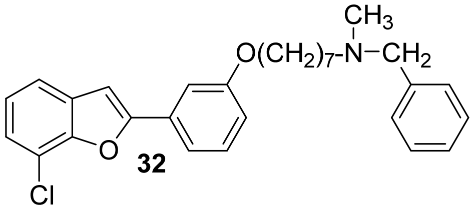
32

Using the previous procedure and starting from benzofuran **27** (0.10 g, 0.24 mmol), *N*-methylbenzylamine (0.06 mL, 0.48 mmol) and NaI (0.03 g, 0.24 mmol) in toluene (40 mL). Purification by column chromatography on silica gel in diethyl ether/acetone 9.8:0.2. 31. **32** was obtained as yellowish oil: (0.06 g, 54% yield); ^{1}H NMR (600 MHz, $CDCl_3$): δ = 7.51 (dd, *J* = 7.7, 1.0 Hz, 1H), 7.47 (ddd, *J* = 7.7, 1.6, 0.9 Hz, 1H), 7.45-7.41 (m, 2H), 7.36 (t, *J* = 7.9 Hz, 1H), 7.33-7.30

(m, 4H), 7.26-7.22 (m, 1H), 7.10 (t, *J* = 7.7 Hz, 1H), 7.06 (s, 1H), 6.92 (ddd, *J* = 8.2, 2.6, 0.9 Hz, 1H), 4.04 (t, *J* = 6.5 Hz, 2H), 3.49 (s, 2H), 2.38 (dd, *J* = 8.4, 6.5 Hz, 2H), 2.19 (s, 3H), 1.86-1.79 (m, 2H), 1.56-1.48 (m, 4H), 1.41-1.34 (m, 4H) ppm; ^{13}C NMR (151 MHz, $CDCl_3$): δ = 159.61, 156.82, 150.69, 139.20, 131.16, 130.91, 129.98, 129.21, 128.28, 127.00, 124.51, 123.91, 119.48, 117.64, 116.74, 115.25, 111.37, 102.05, 68.20, 62.41, 57.56, 42.31, 30.44, 29.83, 29.43, 29.37, 27.47, 27.38, 26.17 ppm; MS (EI, 70eV) *m/z* (%): 461 (7) [$M^{+\cdot}$ (^{35}Cl)], 463 (2) [M+2 (^{37}Cl)], 134 (100) [C_6H_5-CH_2-$N(CH_3)CH_2$]$^+$, 91 (27) [C_7H_7]$^+$.

1.10 7-[3-(5-Chlorobenzofuran-2-yl)-phenoxy]-*N*-benzyl-*N*-methylheptanamine (33)

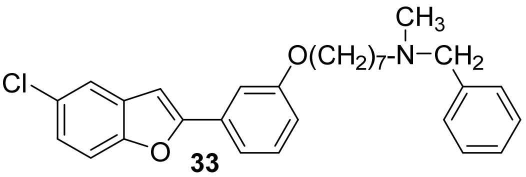

33

Using the previous procedure and starting from benzofuran **28** (0.17 g, 0.40 mmol), *N*-methylbenzylamine (0.10 mL, 0.80 mmol) and NaI (0.06 g, 0.40 mmol) in toluene (60 mL). Purification by column chromatography on silica gel in diethyl ether/acetone 9:1. **33** was obtained as white solid: (0.06 g, 33% yield); mp: 60-62 °C; ^{1}H NMR (600 MHz, $CDCl_3$): δ = 7.70- 7.62 (m, 2H), 7.41 (d, J = 2.0 Hz, 1H), 7.30 (s, 1H), 7.25-7.20 (m, 4H), 7.19-7.13 (m, 1H), 7.10 (dd, *J* = 8.7, 2.2 Hz, 1H), 6.89-6.85 (m, 2H), 6.71 (d, *J* = 0.8 Hz, 1H), 3.91 (t, *J* = 6.5 Hz, 2H), 3.41 (s, 2H), 2.33-2.25 (m, 2H), 2.11 (s, 3H), 1.76-1.66 (m, 2H), 1.46 (m, 2H), 1.42-1.36 (m, 2H), 1.28 (m, 4H) ppm; ^{13}C NMR (151 MHz, $CDCl_3$): δ = 160.03, 157.79, 153.18, 139.17, 131.05, 129.24, 128.46, 128.32, 127.05, 126.68, 123.88, 122.66, 120.18, 114.97, 112.00, 99.17, 68.22, 62.41, 57.55, 42.33, 29.36, 27.43, 26.13 ppm; MS (EI, 70eV) *m/z* (%): 461 (6) [$M^{+\cdot}$ (^{35}Cl)], 463 (2) [M+2 (^{37}Cl)], 134 (100) [C_6H_5-CH_2-$N(CH_3)CH_2$]$^+$, 91 (17) [C_7H_7]$^+$.

1.11 7-[3-(7-Bromobenzofuran-2-yl)-phenoxy]-*N*-benzyl-*N*-methylheptanamine (34)

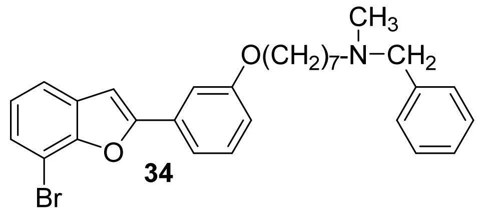

Using the previous procedure and starting from benzofuran **29** (0.15 g, 0.32 mmol), *N*-methylbenzylamine (0.08 mL, 0.64 mmol) and NaI (0.06 g, 0.40 mmol) in toluene (50 mL). Purification by column chromatography on silica gel in diethylether/acetone 9.5:0.5. **34** was obtained as white solid: (0.11 g, 69% yield); mp: 28-30 °C; ^{1}H NMR (600 MHz, $CDCl_3$): δ = 7.51 (dd, *J* = 7.7, 1.1 Hz, 1H), 7.47 (dt, *J* = 7.7, 1.3 Hz, 1H), 7.45-7.41 (m, 2H), 7.36 (t, *J* = 7.9 Hz, 1H), 7.33-7.29 (m, 4H), 7.24 (ddd, *J* = 8.7, 5.4, 3.4 Hz, 1H), 7.10 (t, *J* = 7.8 Hz, 1H), 7.06 (s, 1H), 6.92 (ddd, *J* = 8.2, 2.5, 0.9 Hz, 1H), 4.04 (t, *J* = 6.5 Hz, 2H), 3.49 (s, 2H), 2.38 (dd, *J* = 8.4, 6.5 Hz, 2H), 2.19 (s, 3H), 1.85-1.79 (m, 2H), 1.55 (m, 2H), 1.52-1.47 (m, 2H), 1.42-1.32 (m, 4H) ppm; ^{13}C NMR (151 MHz, $CDCl_3$): δ = 159.49, 156.60, 151.94, 139.11, 131.06, 130.46, 129.89, 129.10, 128.18, 127.27, 126.90, 124.20, 120.01, 117.56, 115.10, 111.32, 103.94, 102.08, 68.10, 62.31, 57.46, 42.23, 29.33, 29.26, 27.36, 27.29, 26.05 ppm; HRMS (ESI): calcd. for $C_{29}H_{33}BrNO_2$ $[M+H]^+$ = 506.1695; found 506.1692.

1.12 7-[3-(5-Bromobenzofuran-2-yl)-phenoxy]-*N*-benzyl-*N*-methylheptanamine (35)

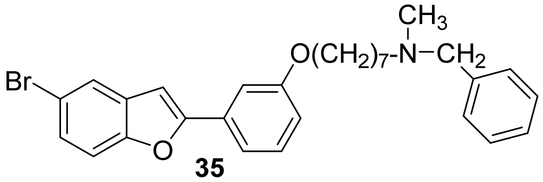

Using the previous procedure and starting from benzofuran **30** (0.38 g, 0.80 mmol), *N*-methylbenzylamine (0.21 mL, 0.016 mmol) and NaI (0.12 g, 0.80 mmol) in toluene (50 mL). Purification by column chromatography on silica gel in diethylether/acetone 9.5:0.5. **35** was obtained as white solid: (0.30 g, 76% yield); mp: 35-37 °C; ^{1}H NMR (600 MHz, $CDCl_3$): δ = δ 7.71-7.68 (m, 1H), 7.42 (dt, *J* = 7.7, 1.2 Hz, 1H), 7.40-7.37 (m, 3H), 7.36-7.33 (m, 1H), 7.33-7.29 (m, 4H), 7.24

(ddd, J = 8.5, 3.8, 2.6 Hz, 1H), 6.94 (d, J = 0.7 Hz, 1H), 6.91 (ddd, J = 8.2, 2.6, 1.0 Hz, 1H), 4.03 (t, J = 6.5 Hz, 2H), 3.49 (s, 2H), 2.40-2.36 (m, 2H), 2.20 (s, 3H), 1.82 (dq, J = 8.5, 6.5 Hz, 2H), 1.60-1.47 (m, 4H), 1.37 (m, 4H) ppm; ^{13}C NMR (151 MHz, $CDCl_3$): δ = 159.52, 157.16, 153.56, 139.08, 131.18, 131.11, 129.90, 129.10, 128.18, 127.10, 126.91, 123.47, 117.45, 115.98, 115.36, 112.59, 110.99, 100.86, 68.09, 62.30, 57.45, 42.22, 29.31, 29.24, 27.35, 27.28, 26.04 ppm; HRMS (ESI): calcd. for $C_{29}H_{33}BrNO_2$ $[M+H]^+$ = 506.1695; found 506.1703.

2. NMR and Mass Spectra

^{1}H NMR spectra of **2-[3-(7-bromoheptyloxy)-phenyl]-benzofuran (26)**

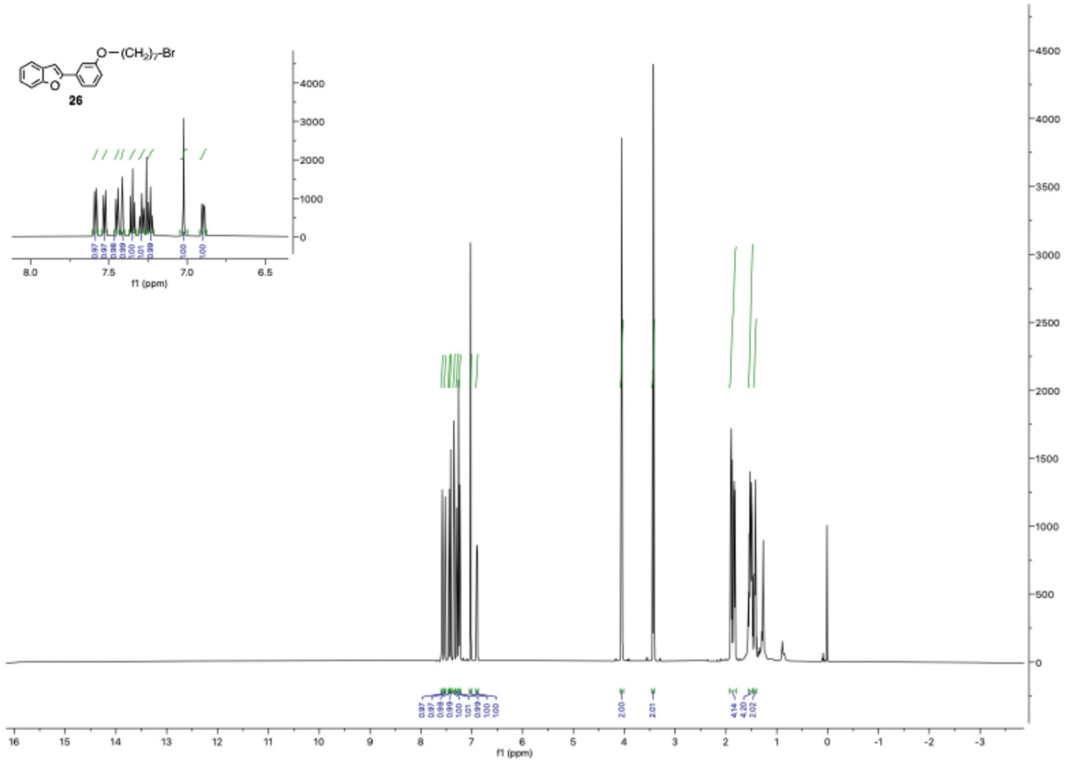

^{13}C NMR spectra of **2-[3-(7-bromoheptyloxy)-phenyl]-benzofuran (26)**

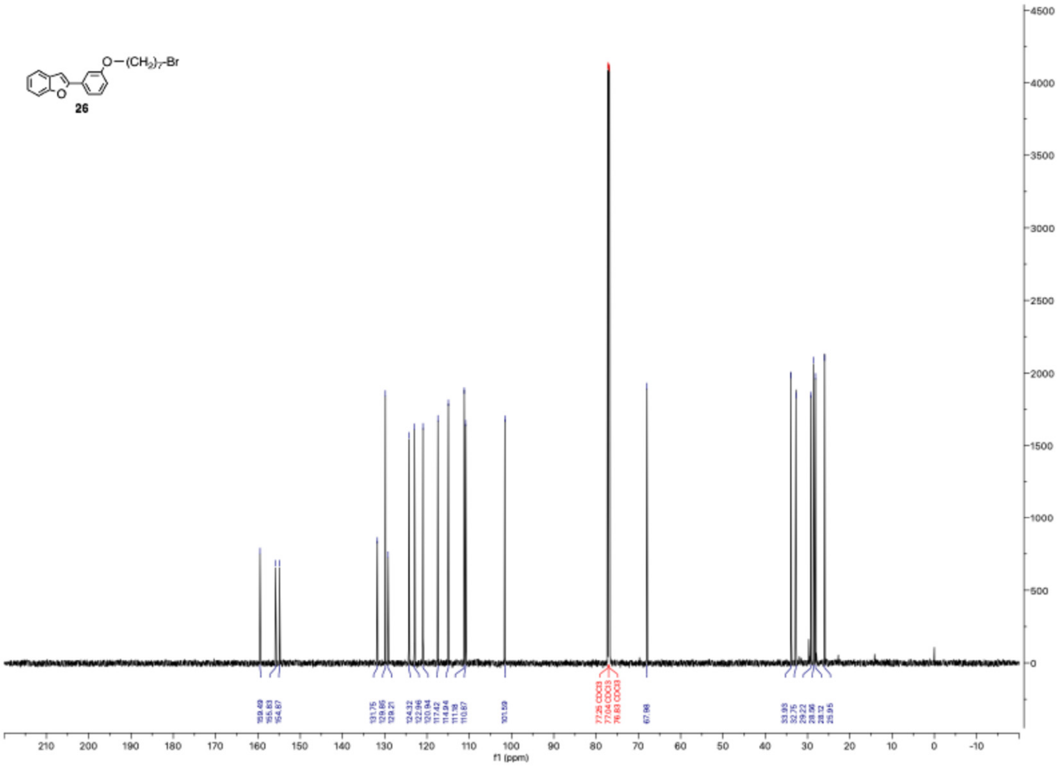

HRMS (ESI) spectra of **2-[3-(7-bromoheptyloxy)-phenyl]-benzofuran (26)**

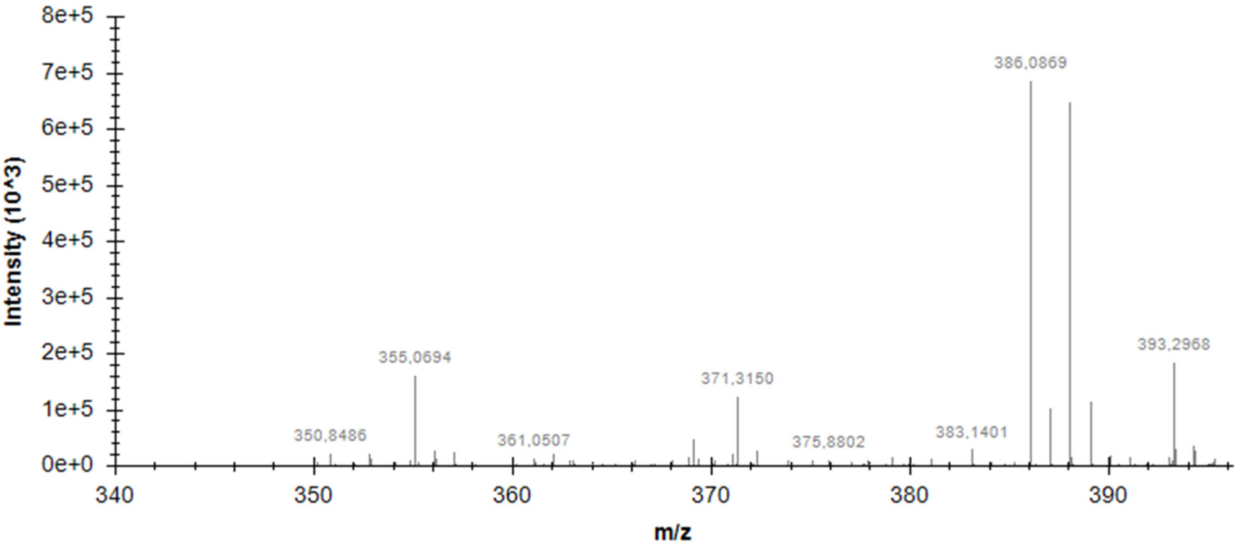

^{1}H NMR spectra of **7-chloro-2-[3-(7-bromoheptyloxy)-phenyl]-benzofuran (27)**

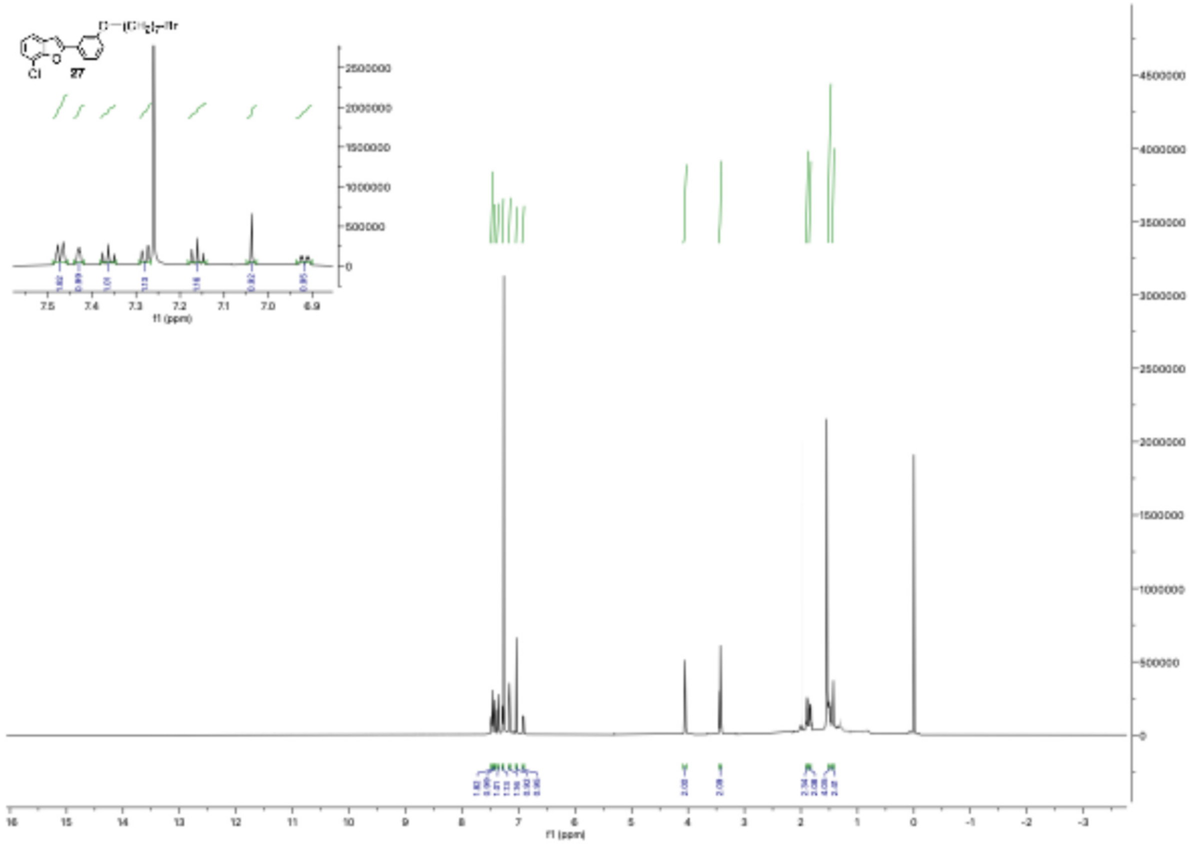

^{13}C NMR spectra of **7-chloro-2-[3-(7-bromoheptyloxy)-phenyl]-benzofuran (27)**

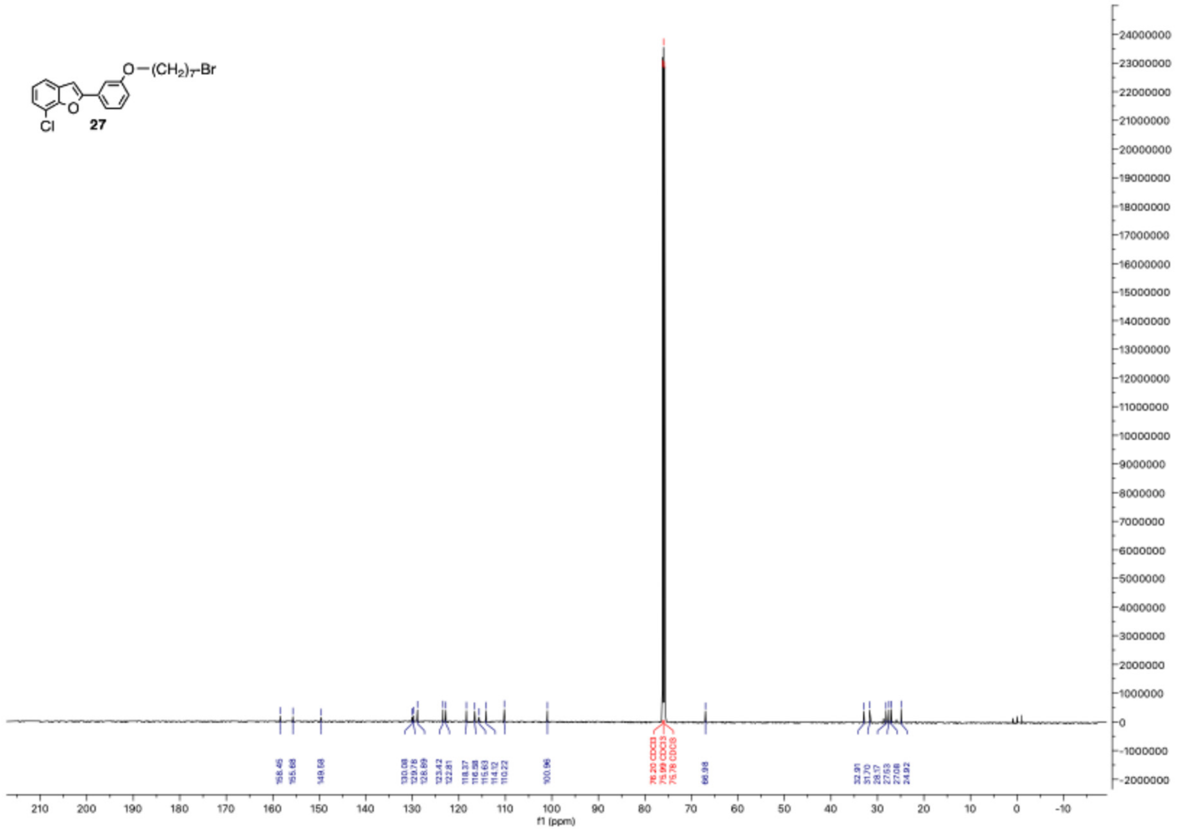

EI (70 eV) mass spectrum of **7-chloro-2-[3-(7-bromoheptyloxy)-phenyl]-benzofuran (27)**

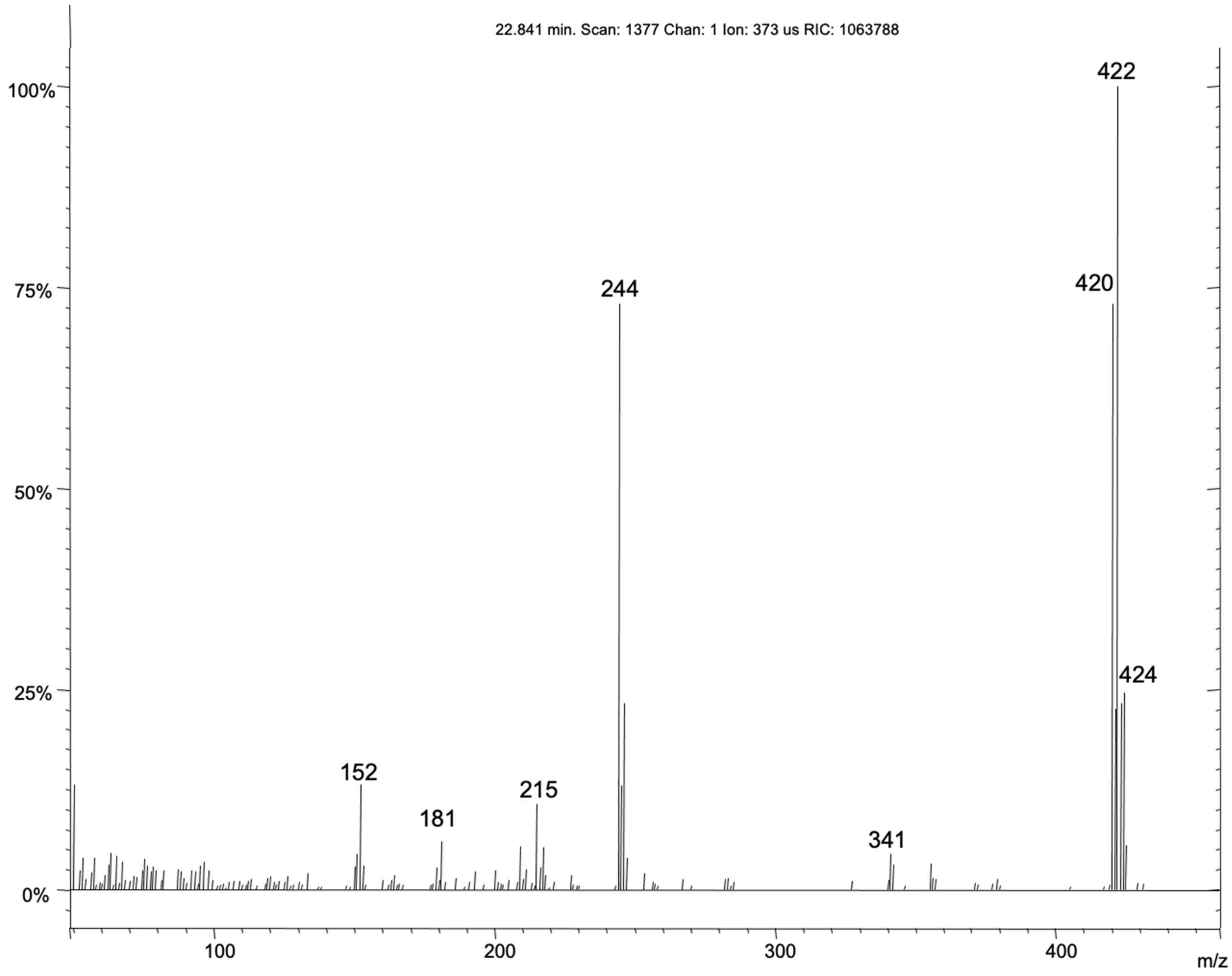

^{1}H NMR spectra of **5-chloro-2-[3-(7-bromoheptyloxy)-phenyl]-benzofuran (28)**

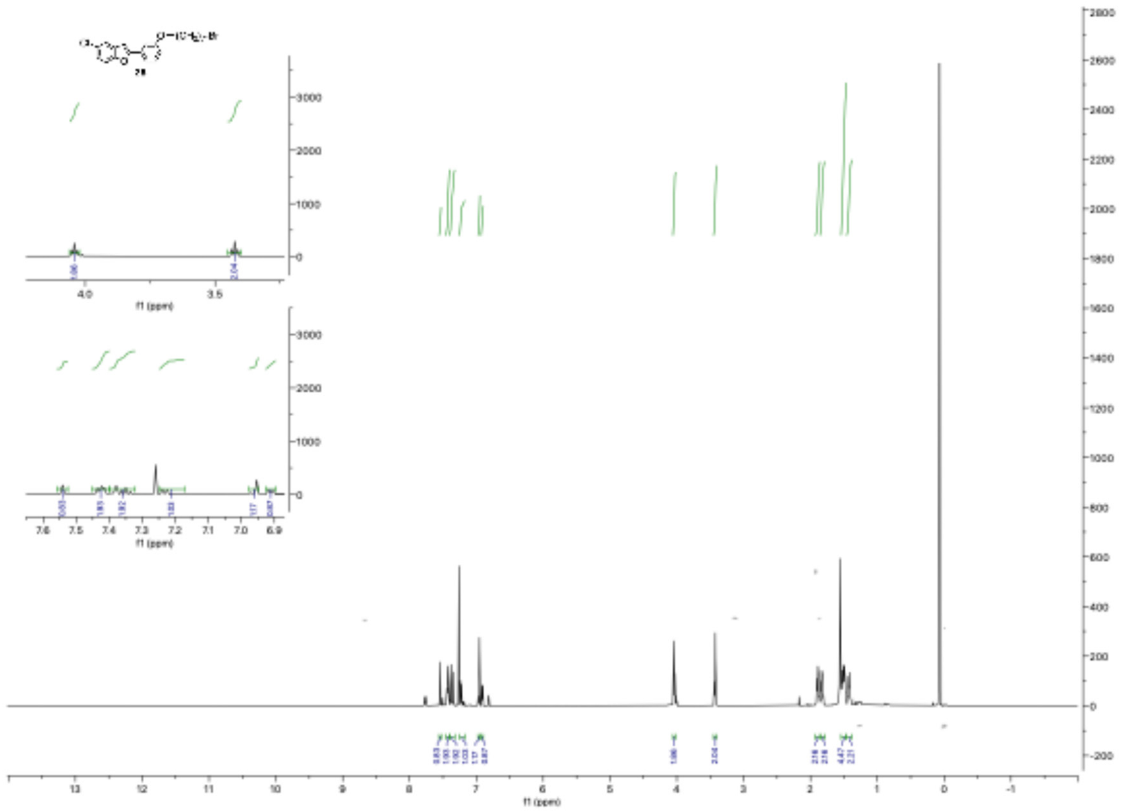

^{13}C NMR spectra of **5-chloro-2-[3-(7-bromoheptyloxy)-phenyl]-benzofuran (28)**

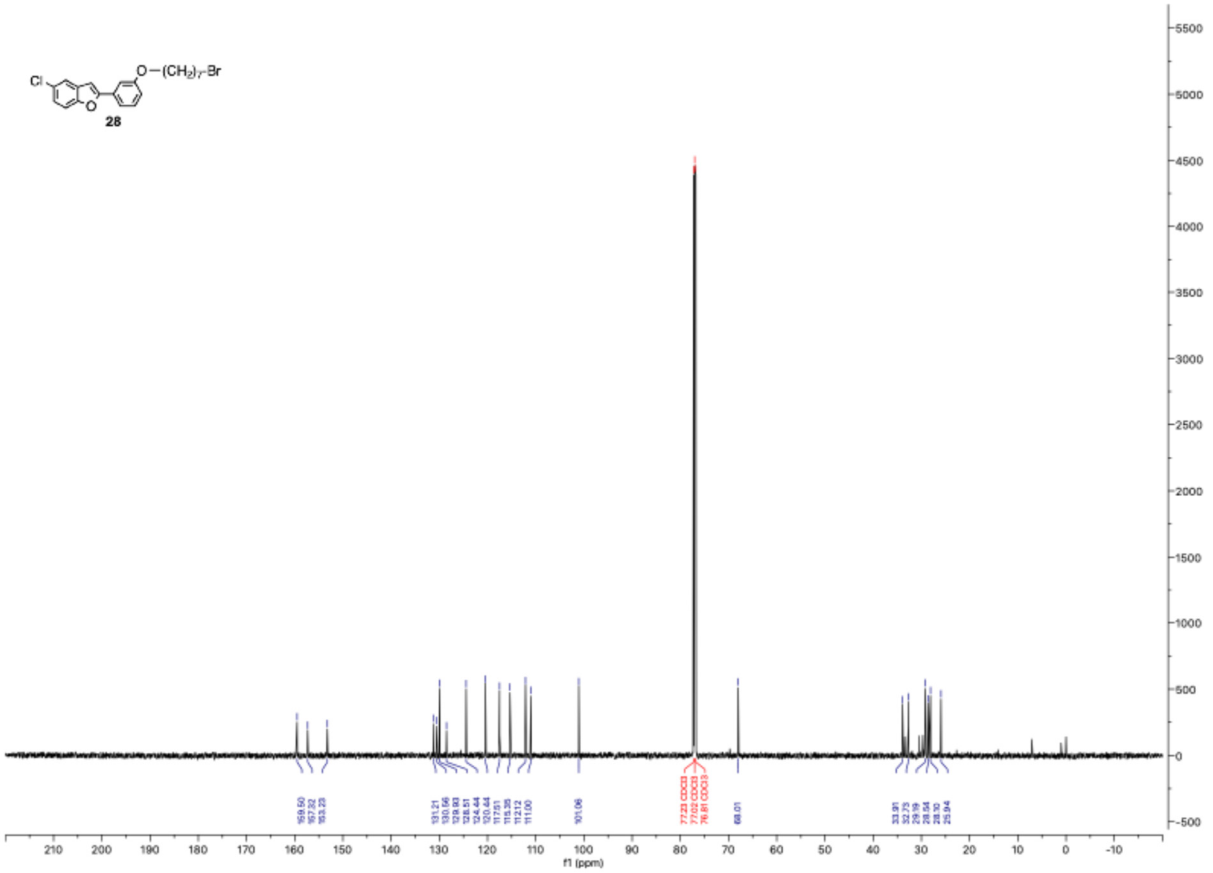

EI (70 eV) mass spectrum of **5-chloro-2-[3-(7-bromoheptyloxy)-phenyl]-benzofuran (28)**

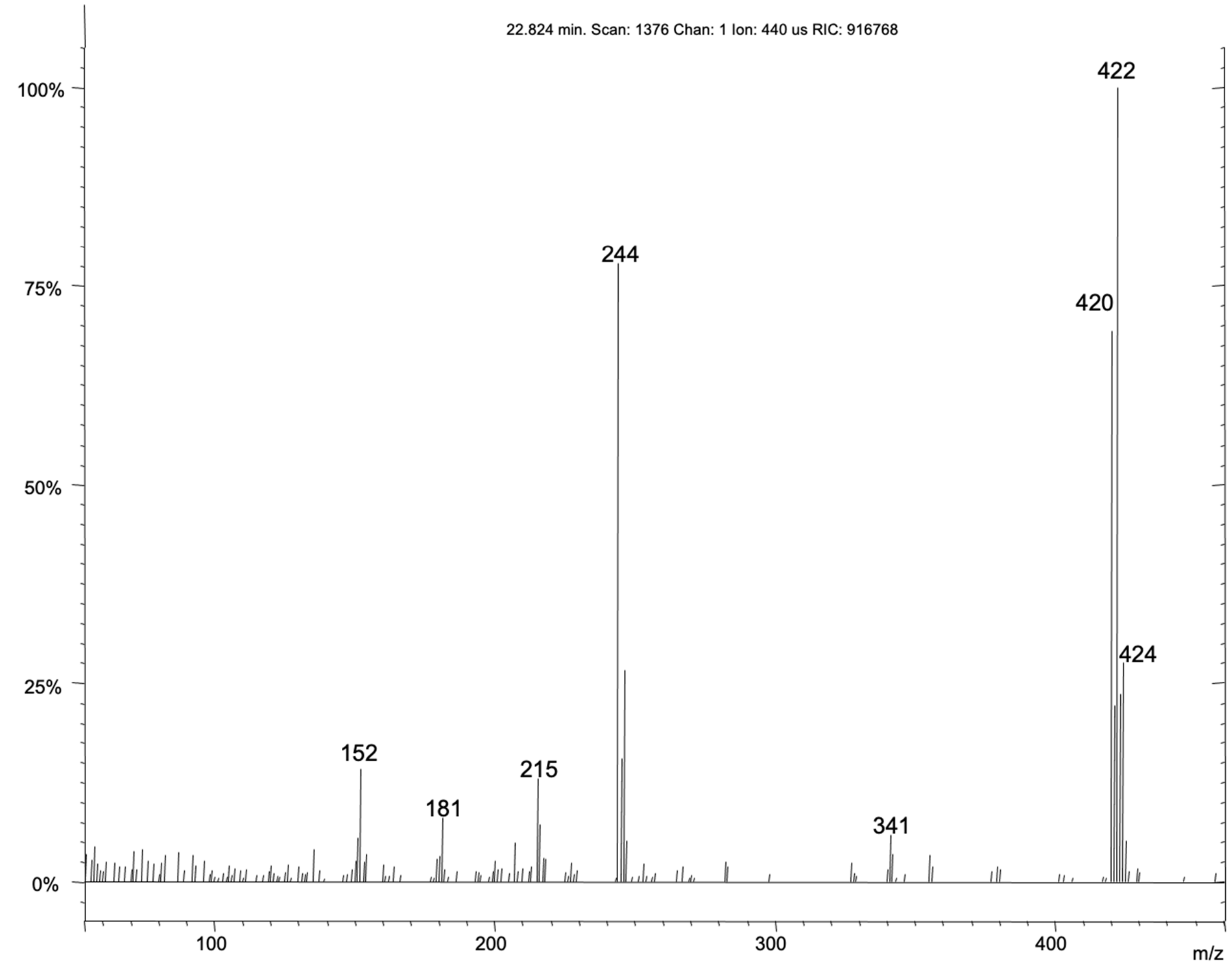

^{1}H NMR spectra of **7-bromo-2-[3-(7-bromoheptyloxy)-phenyl]-benzofuran (29)**

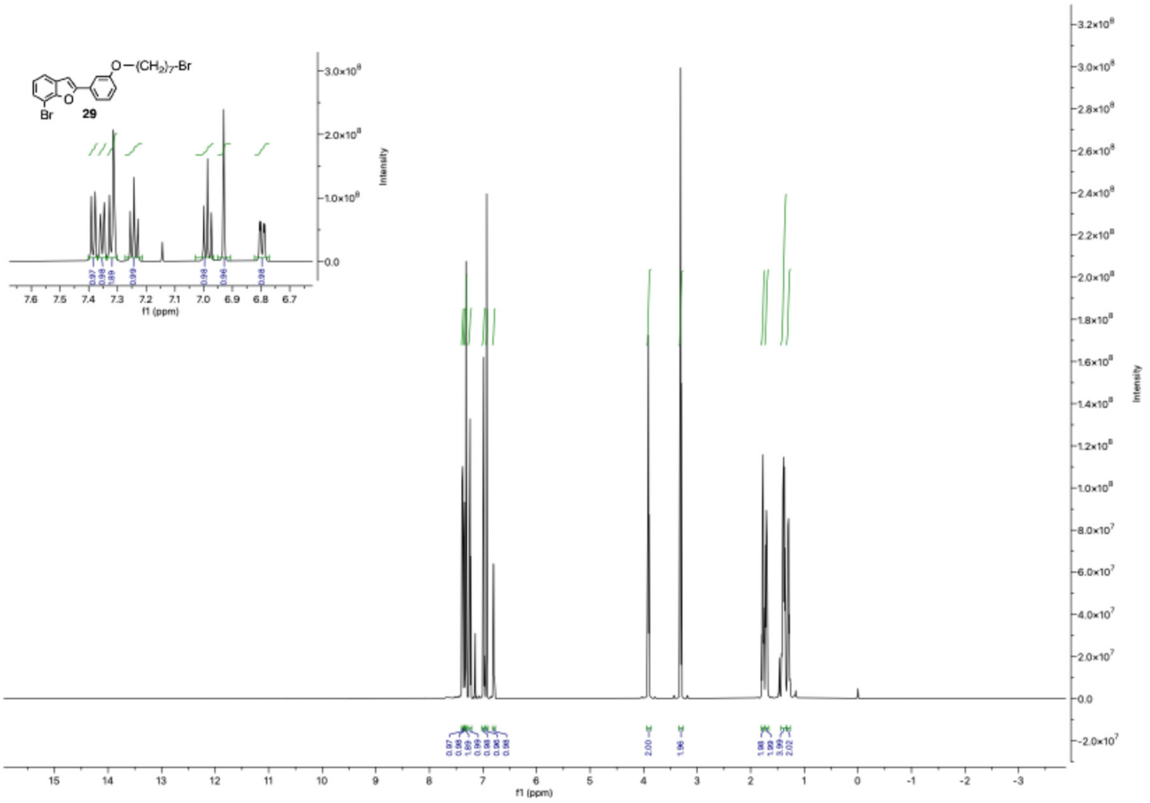

^{13}C NMR spectra of **7-bromo-2-[3-(7-bromoheptyloxy)-phenyl]-benzofuran (29)**

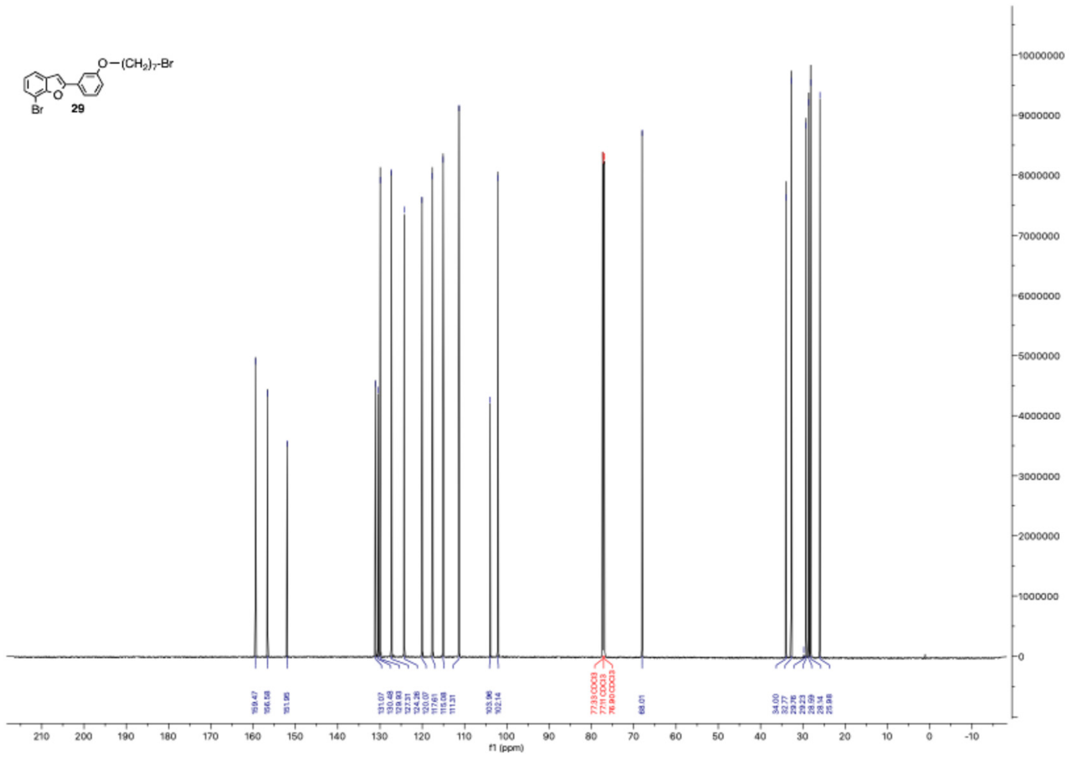

EI (70 eV) mass spectrum of **7-bromo-2-[3-(7-bromoheptyloxy)-phenyl]-benzofuran (29)**

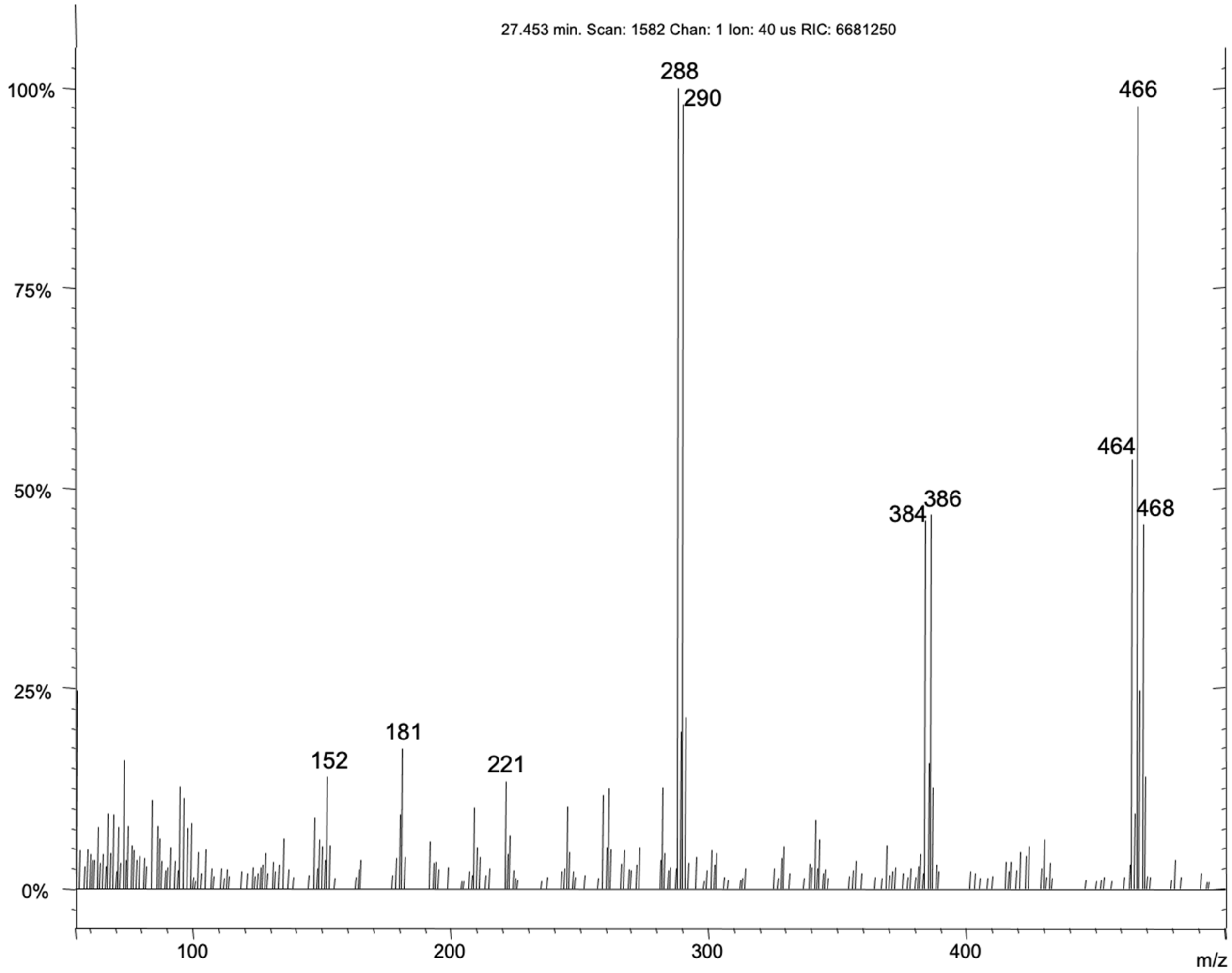

^{1}H NMR spectra of **5-bromo-2-[3-(7-bromoheptyloxy)-phenyl]-benzofuran (30)**

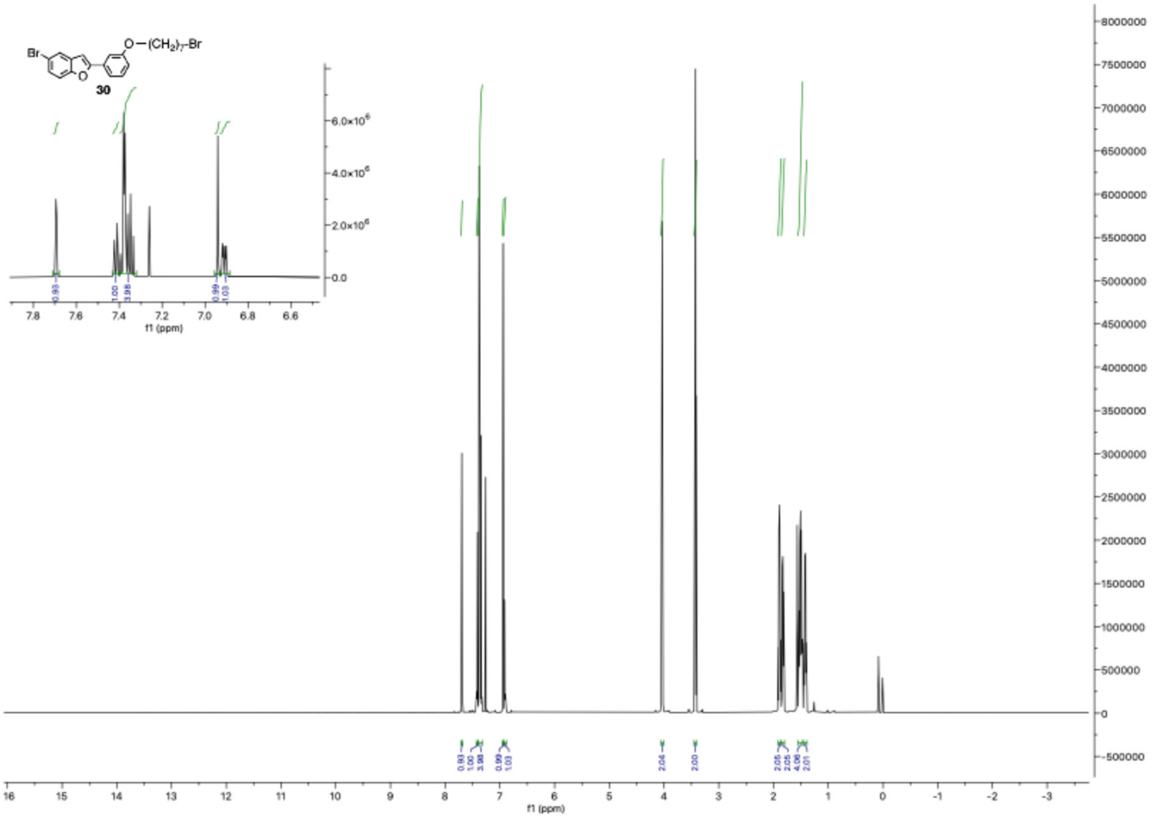

^{13}C NMR spectra of **5-bromo-2-[3-(7-bromoheptyloxy)-phenyl]-benzofuran (30)**

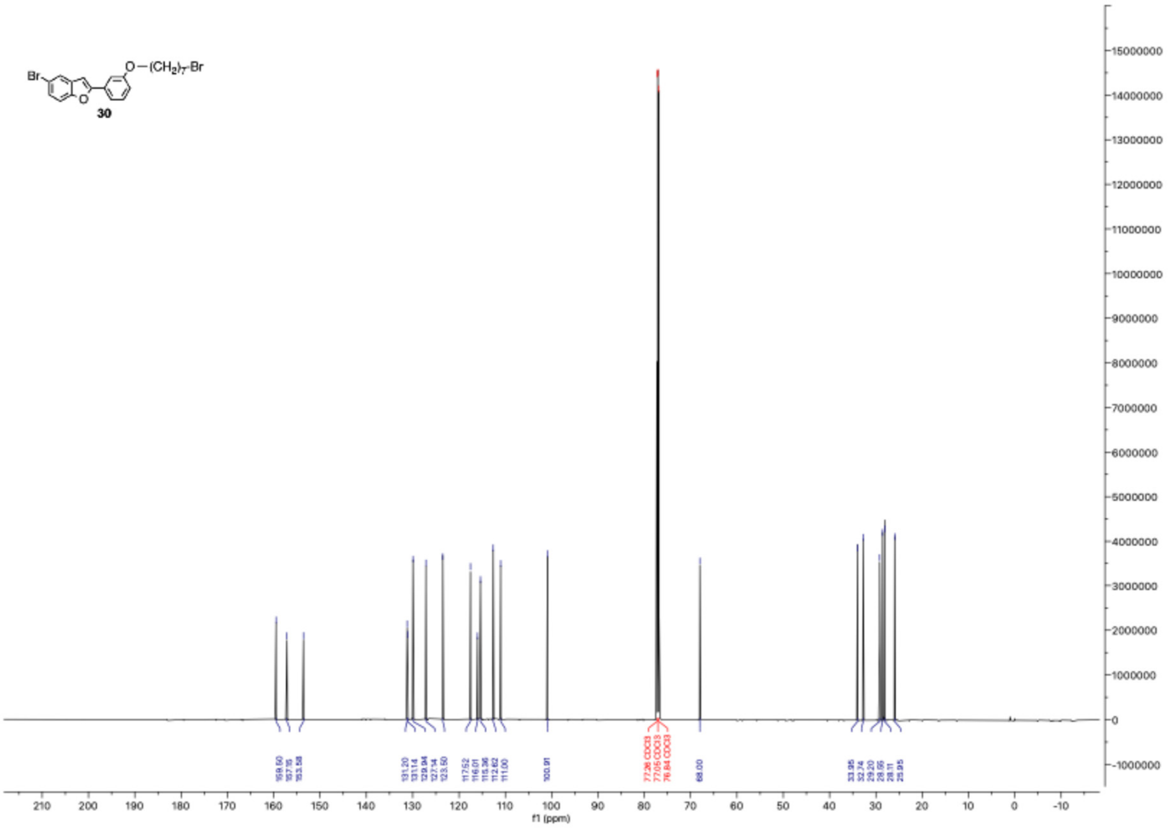

EI (70 eV) mass spectrum of **5-bromo-2-[3-(7-bromoheptyloxy)-phenyl]-benzofuran (30)**

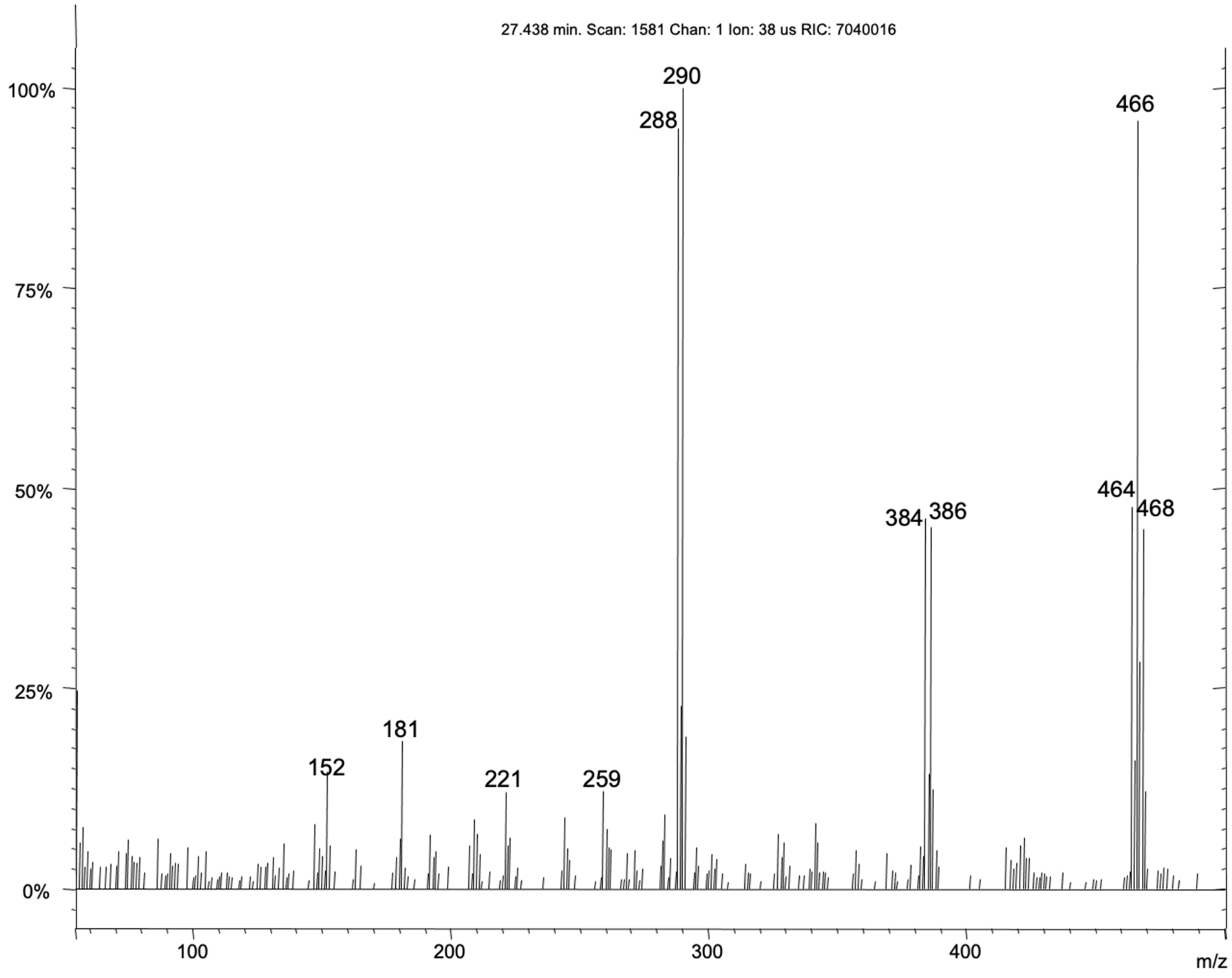

^{1}H NMR spectra of **7-[3-(benzofuran-2-yl)-phenoxy]-*N*-benzyl-*N*-methylheptanamine (31)**

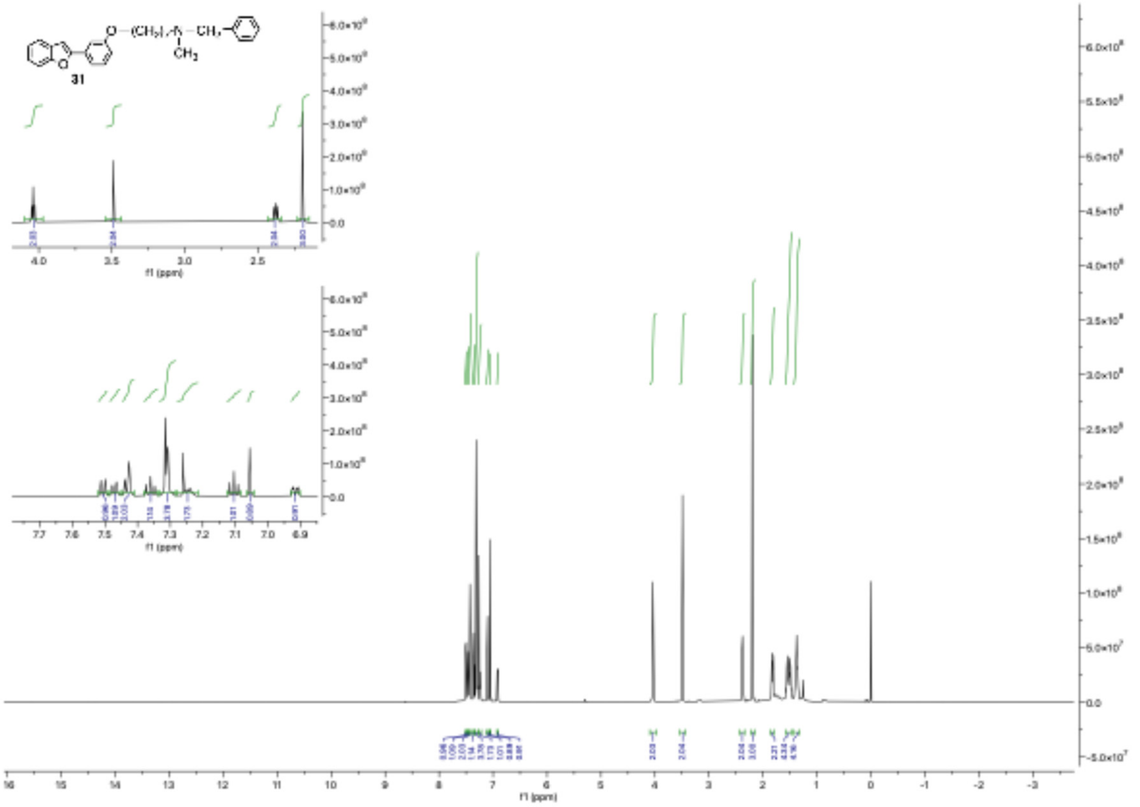

^{13}C NMR spectra of **7-[3-(benzofuran-2-yl)-phenoxy]-*N*-benzyl-*N*-methylheptanamine (31)**

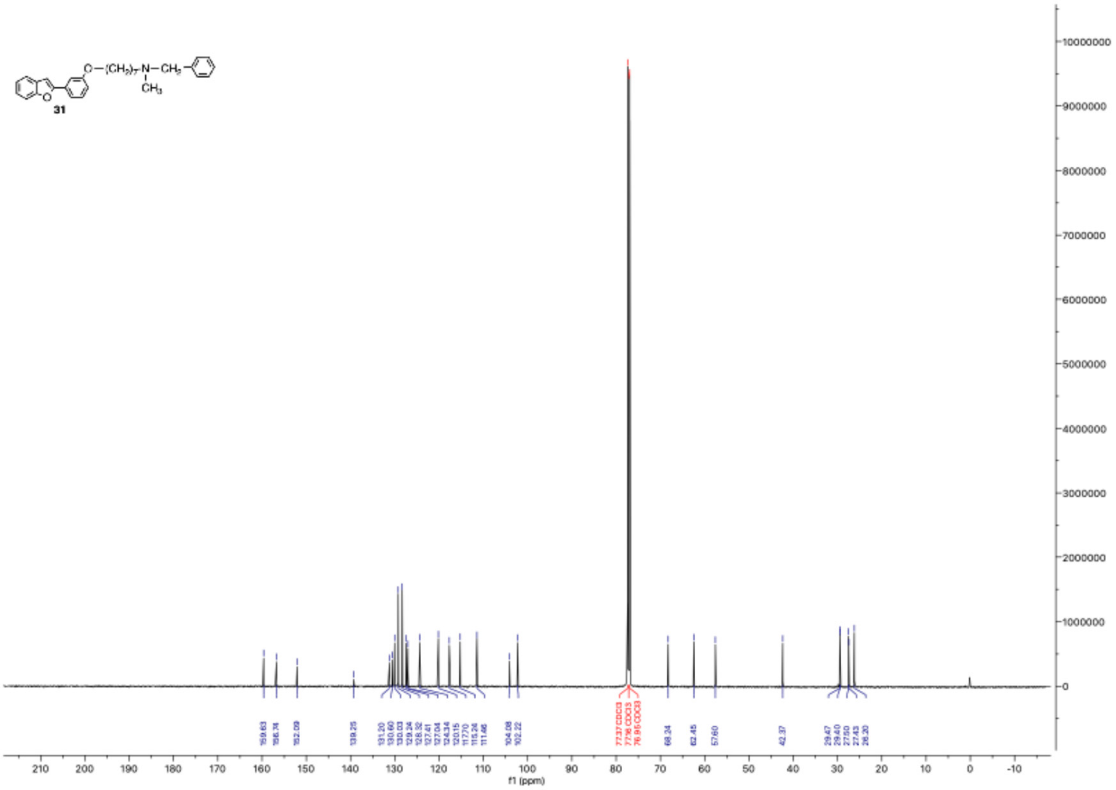

HRMS (ESI) spectra of **7-[3-(benzofuran-2-yl)-phenoxy]-*N*-benzyl-*N*-methylheptanamine (31)**

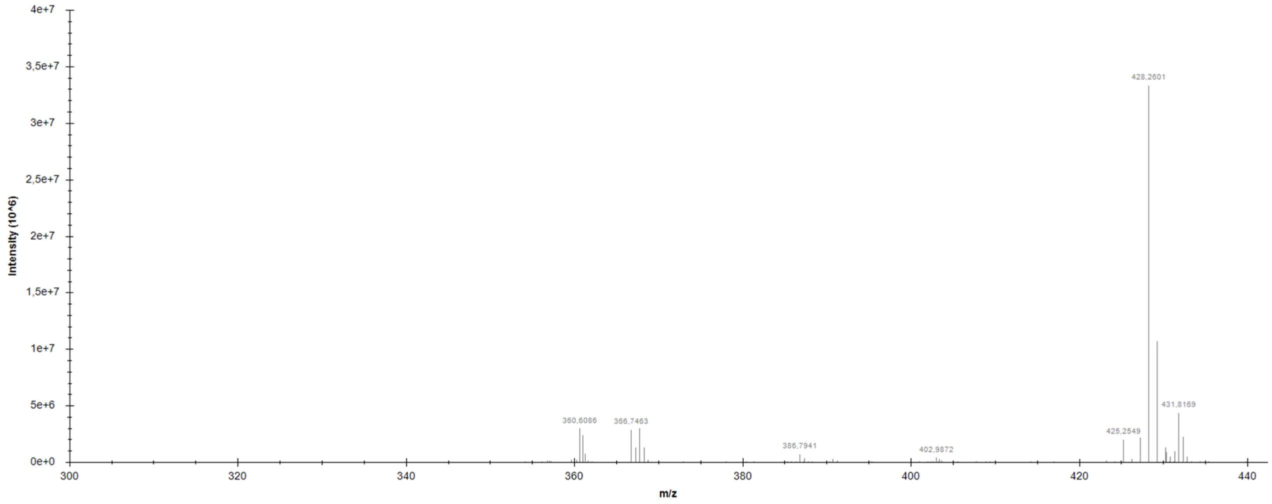

^{1}H NMR spectra of **7-[3-(7-chlorobenzofuran-2-yl)-phenoxy]-*N*-benzyl-*N*-methylheptanamine (32)**

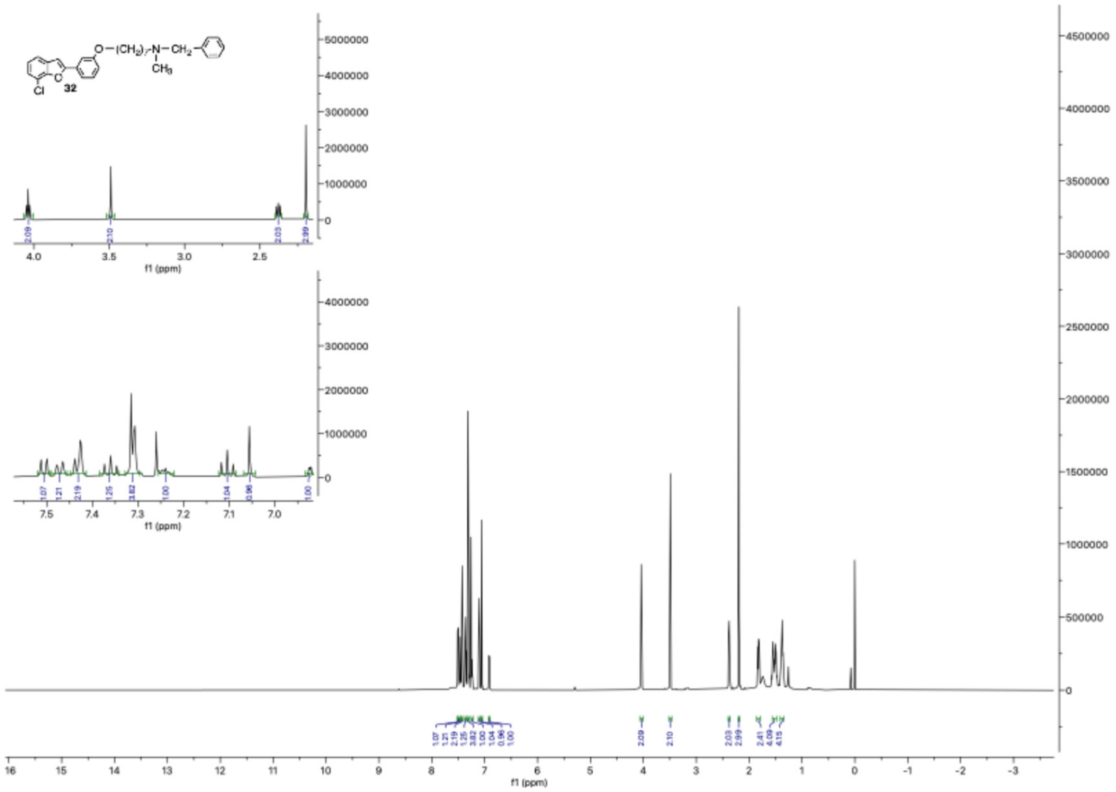

^{13}C NMR spectra of **7-[3-(7-chlorobenzofuran-2-yl)-phenoxy]-*N*-benzyl-*N*-methylheptanamine (32)**

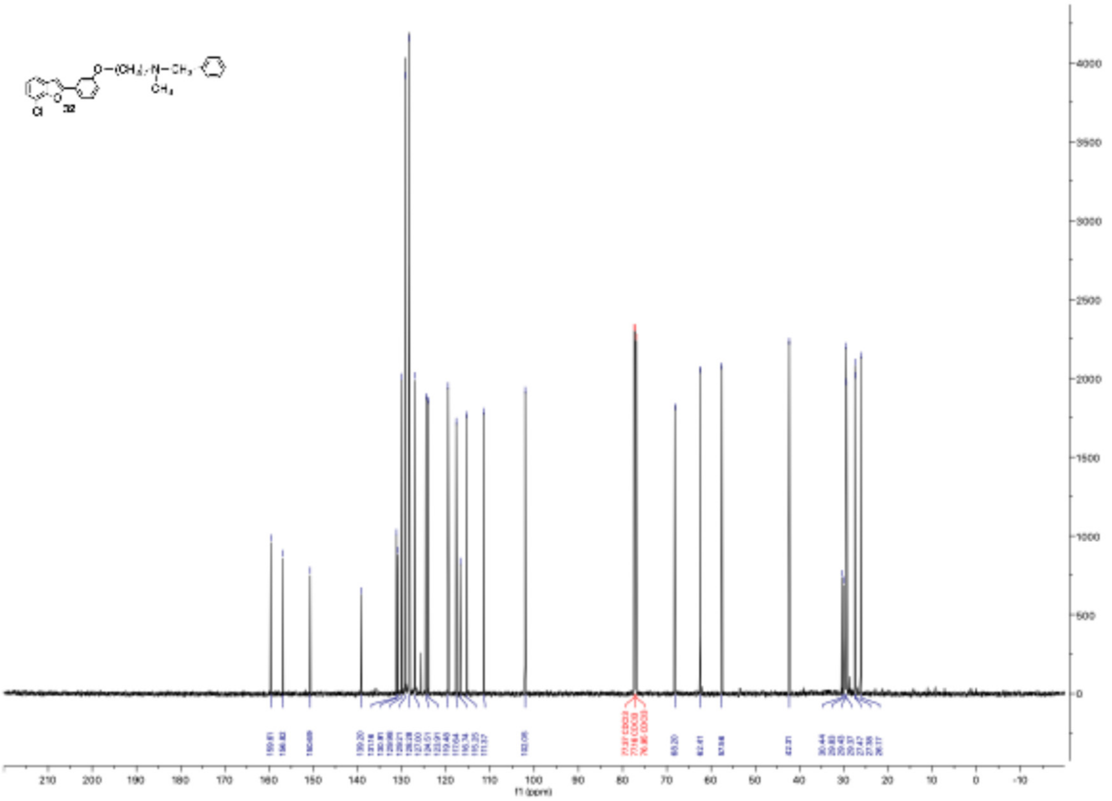

EI (70 eV) mass spectrum of **7-[3-(7-chlorobenzofuran-2-yl)-phenoxy]-*N*-benzyl-*N*-methylheptanamine (32)**

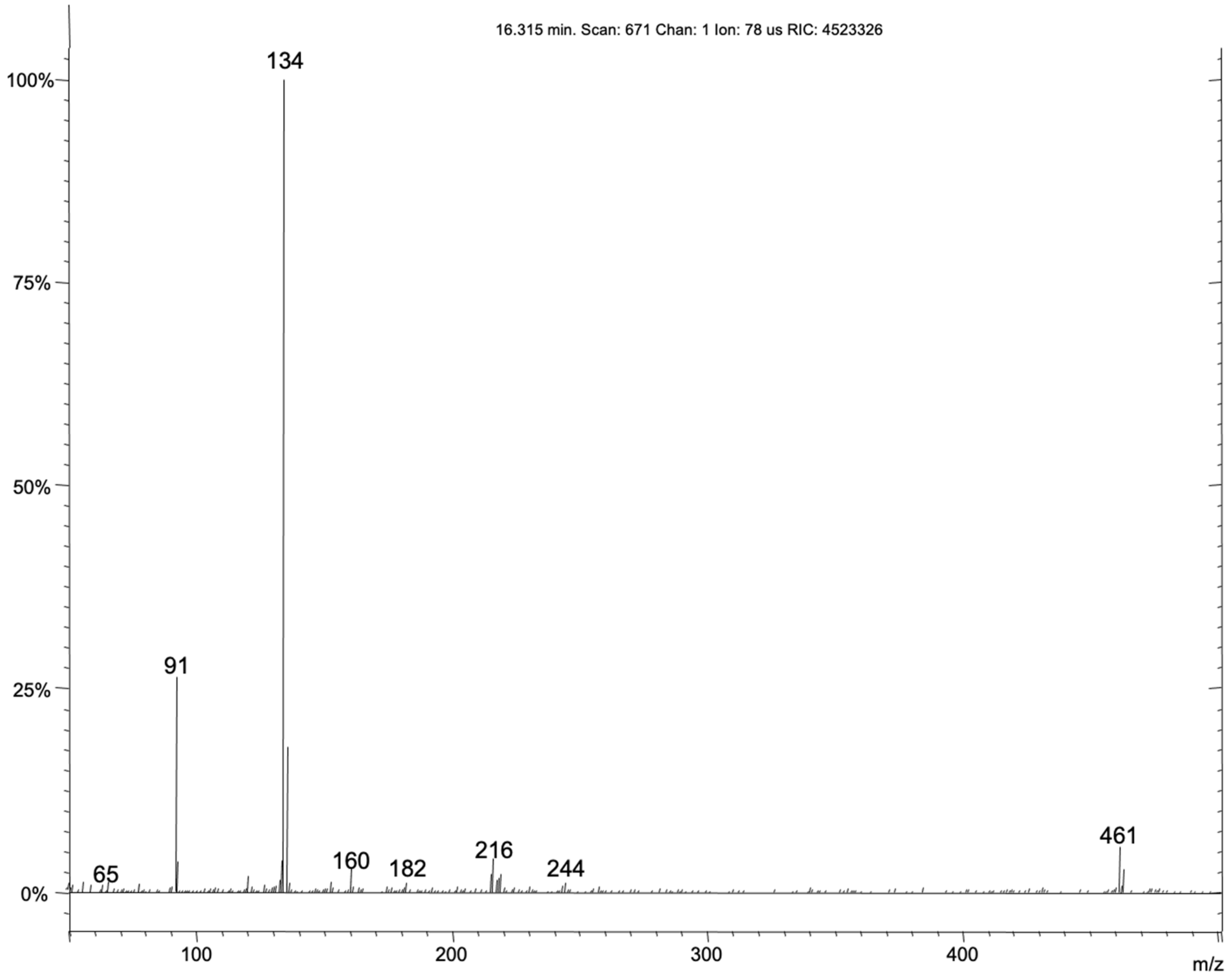

^{1}H NMR spectra of **7-[3-(5-chlorobenzofuran-2-yl)-phenoxy]-*N*-benzyl-*N*-methylheptanamine (33)**

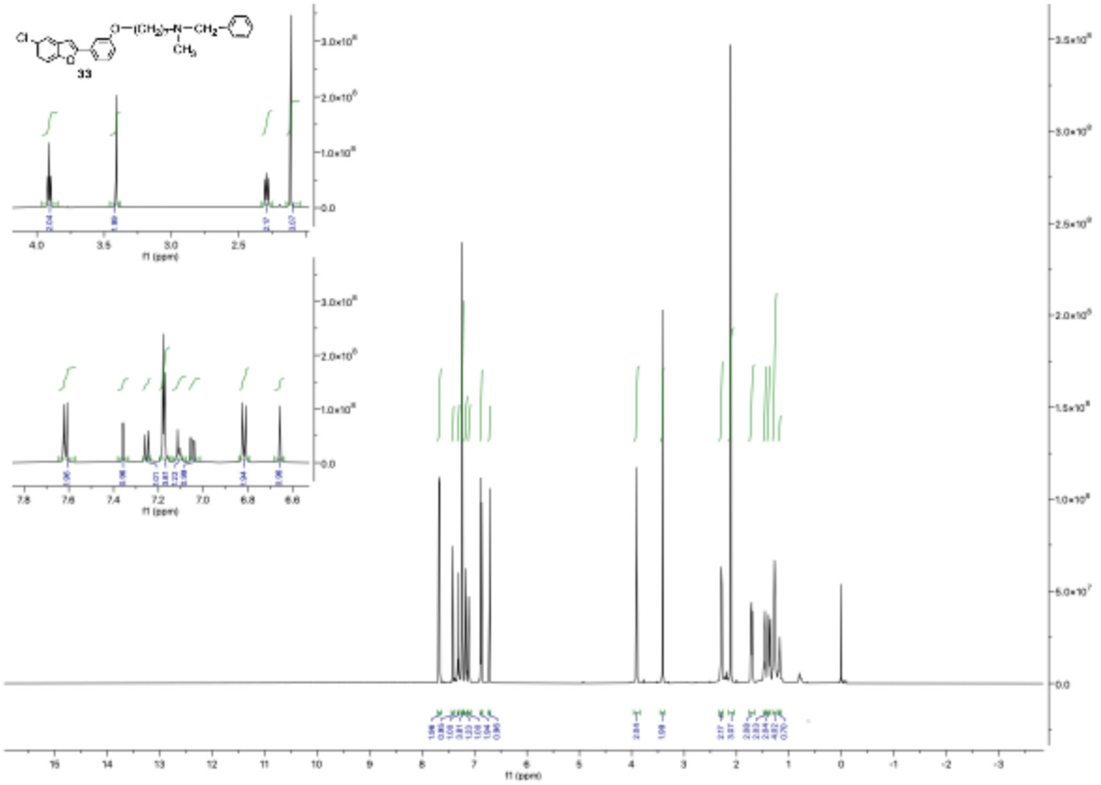

^{13}C NMR spectra of **7-[3-(5-chlorobenzofuran-2-yl)-phenoxy]-*N*-benzyl-*N*-methylheptanamine (33)**

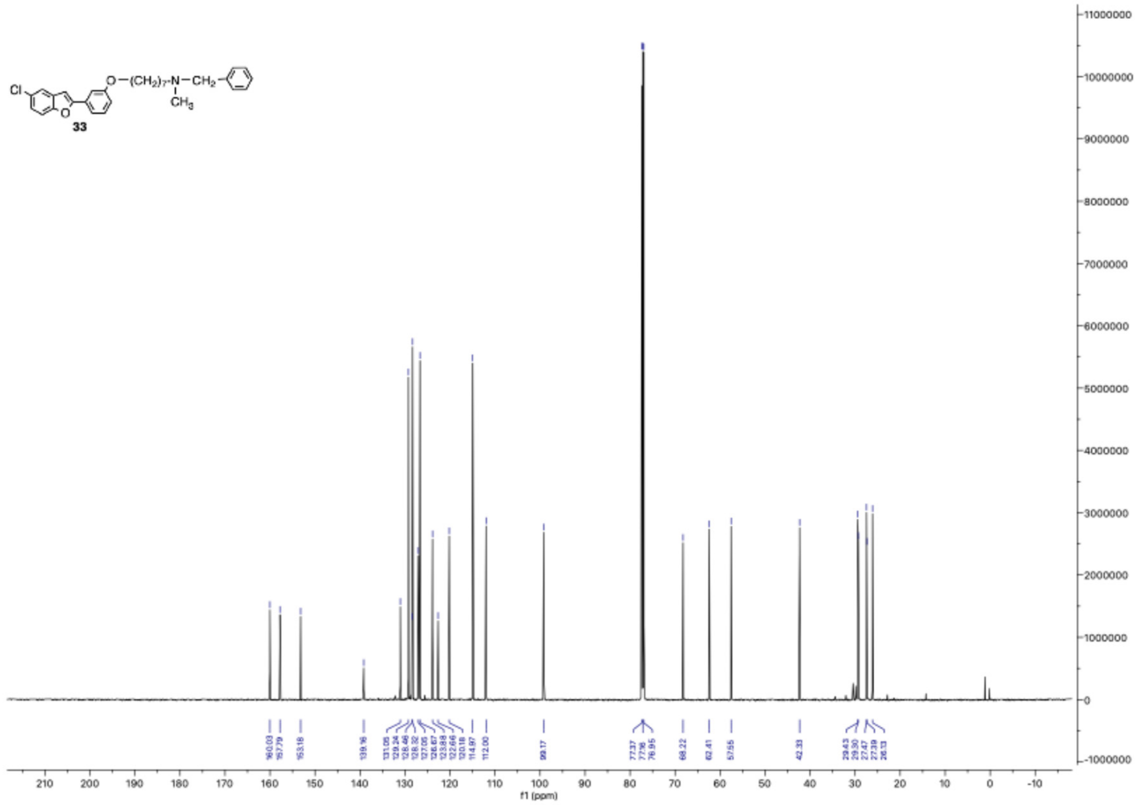

EI (70 eV) mass spectrum of **7-[3-(5-chlorobenzofuran-2-yl)-phenoxy]-*N*-benzyl-*N*-methylheptanamine (33)**

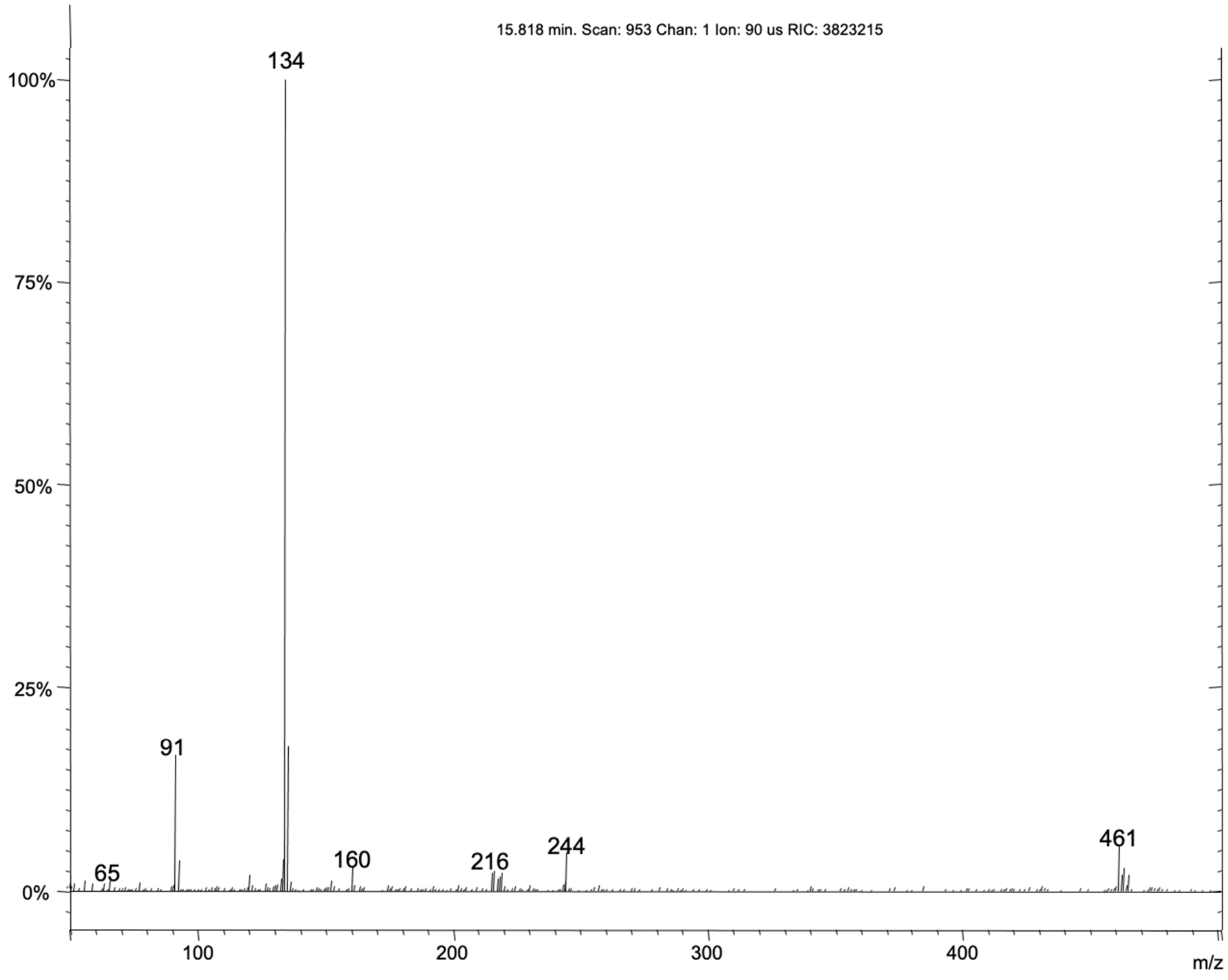

^{1}H NMR spectra of **7-[3-(7-bromobenzofuran-2-yl)-phenoxy]-*N*-benzyl-*N*-methylheptanamine (34)**

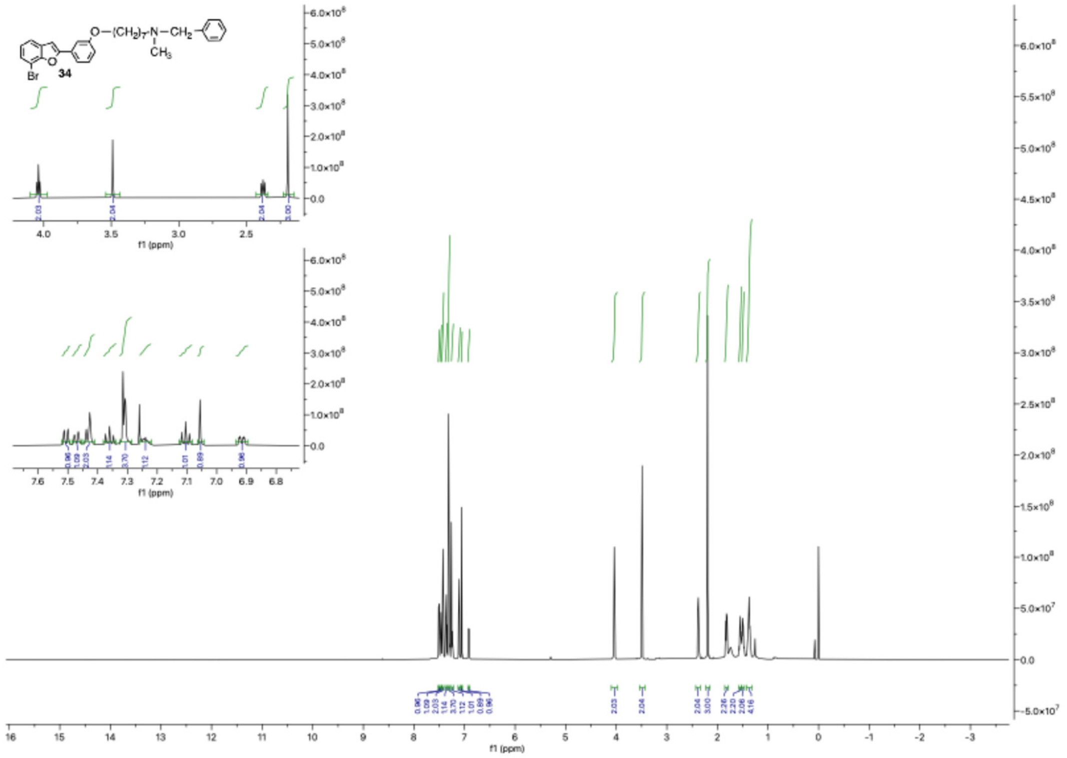

^{13}C NMR spectra of **7-[3-(7-bromobenzofuran-2-yl)-phenoxy]-*N*-benzyl-*N*-methylheptanamine (34)**

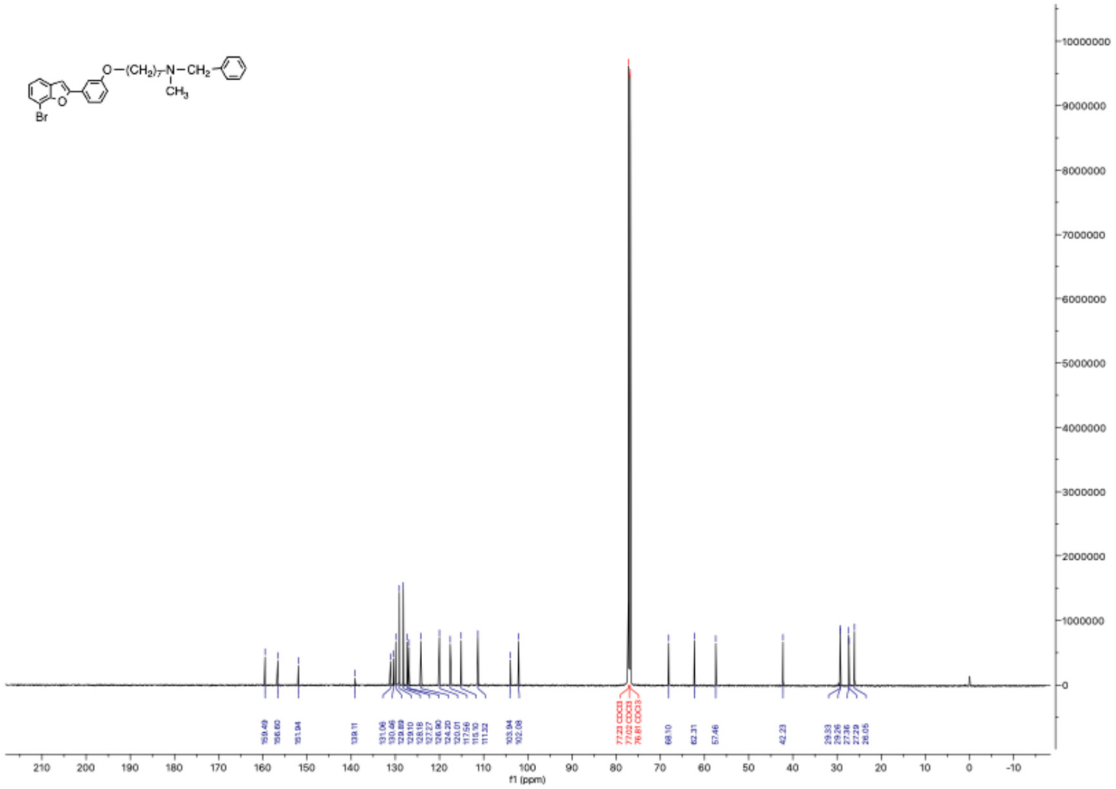

HRMS (ESI) spectra of **7-[3-(7-bromobenzofuran-2-yl)-phenoxy]-*N*-benzyl-*N*-methyl heptanamine (34)**

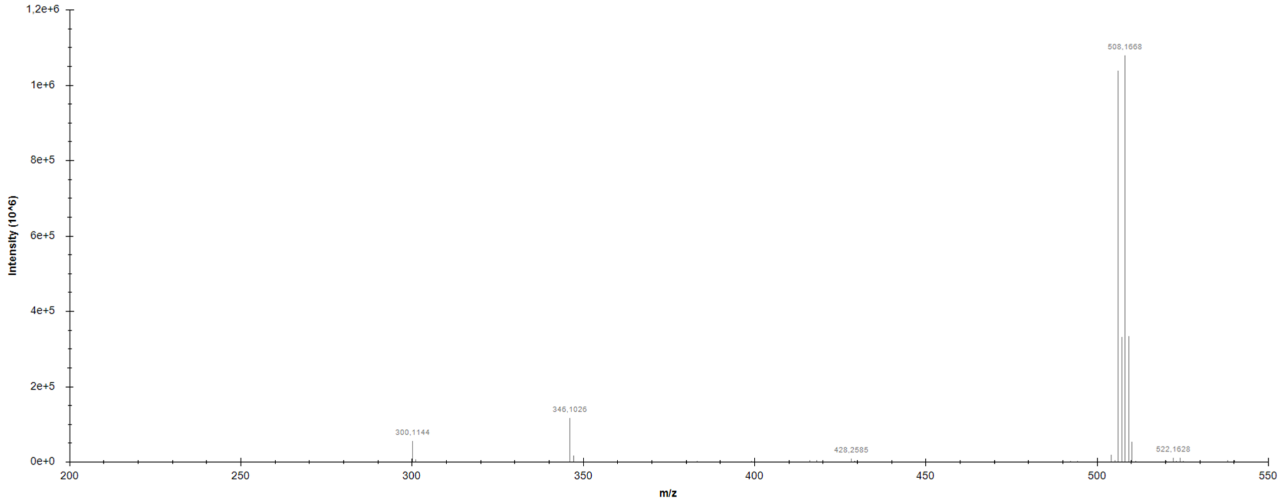

^{1}H NMR spectra of **7-[3-(5-bromobenzofuran-2-yl)-phenoxy]-*N*-benzyl-*N*-methylheptanamine (35)**

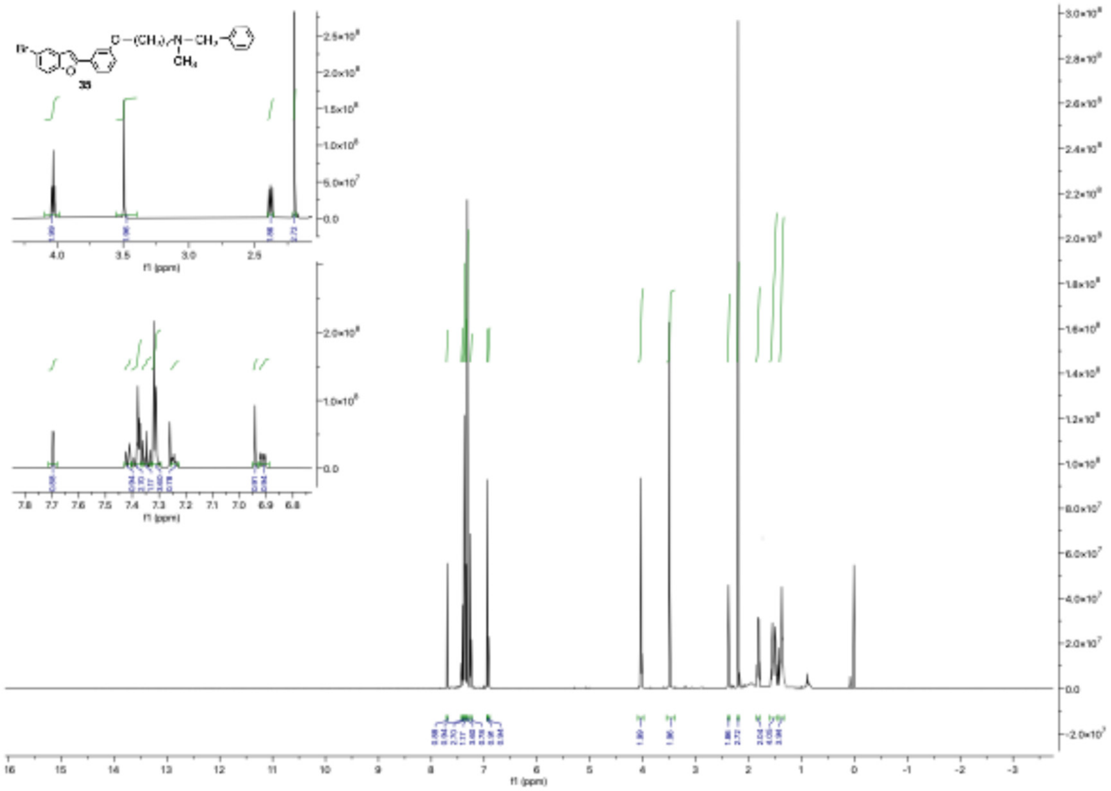

^{13}C NMR spectra of **7-[3-(5-bromobenzofuran-2-yl)-phenoxy]-*N*-benzyl-*N*-methylheptanamine (35)**

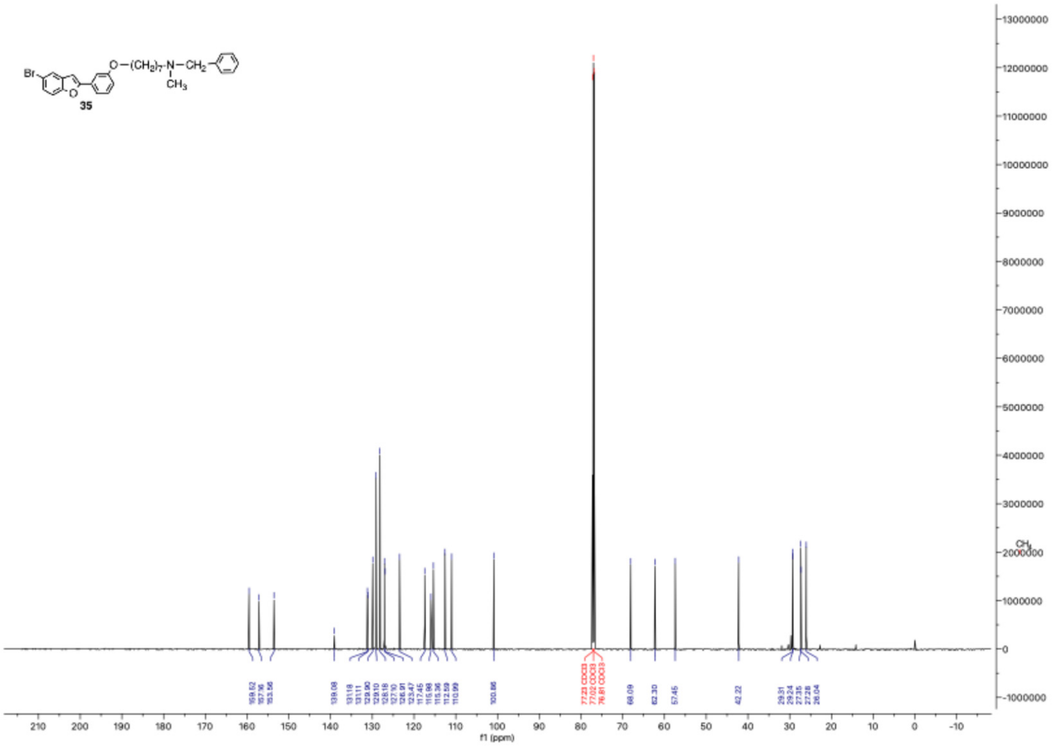

HRMS (ESI) spectra of **7-[3-(5-bromobenzofuran-2-yl)-phenoxy]-*N*-benzyl-*N*-methyl heptanamine (35)**

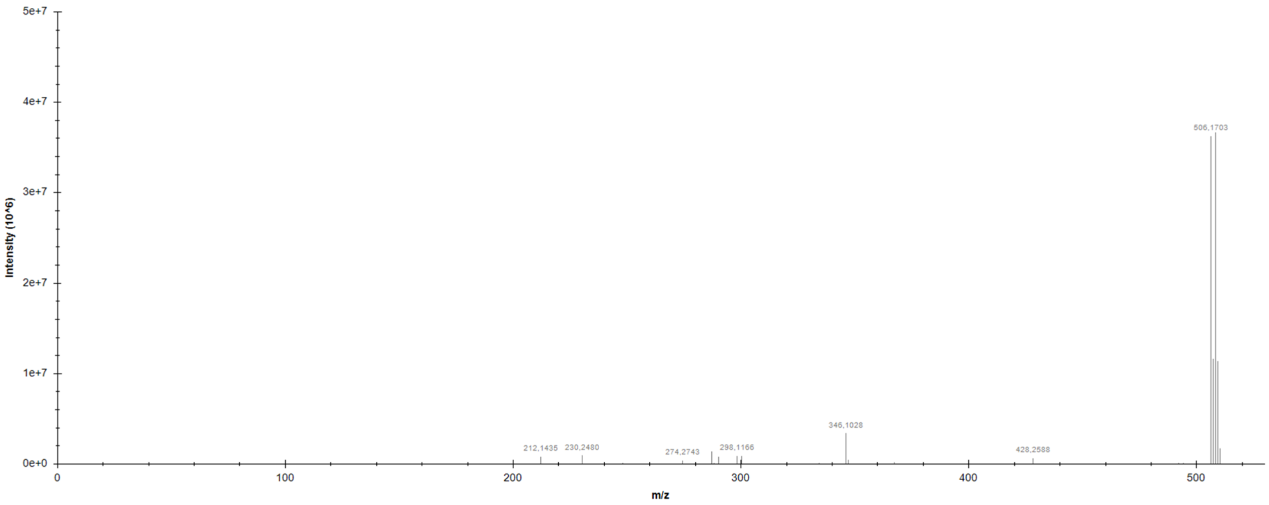

3. Molecular Docking

Table S1. Docking energies and calculated binding affinity of compound **24** with AChE.

Model	Calculated affinity (kcal/mol)
1	-9.390
2	-9.114
3	-8.653
4	-8.452
5	-8.443
6	-8.260
7	-8.086
8	-7.942
9	-7.820
10	-7.728
11	-7.639
12	-7.596
13	-7.487
14	-7.454
15	-7.443
16	-7.358
17	-7.343
18	-7.314
19	-7.296
20	-7.282

Table S2. Docking energies and calculated binding affinity of compound **24** with BChE.

Model	Calculated affinity (kcal/mol)
1	-8.382
2	-8.357
3	-8.342
4	-8.210
5	-7.988
6	-7.977
7	-7.890
8	-7.818
9	-7.816
10	-7.643
11	-7.619
12	-7.531
13	-7.296
14	-7.278
15	-7.277
16	-7.241
17	-7.180
18	-7.157
19	-7.141
20	-7.118

Table S3. Docking energies and calculated binding affinity of compound **29** with AChE.

Model	Calculated affinity (kcal/mol)
1	-8.597
2	-8.401
3	-8.306
4	-8.001
5	-7.957
6	-7.739
7	-7.672
8	-7.430
9	-6.821
10	-6.796
11	-6.693
12	-6.608
13	-6.362
14	-6.099
15	-5.915
16	-5.836
17	-5.682
18	-5.618

Table S4. Docking energies and calculated binding affinity of compound **29** with BChE.

Model	Calculated affinity (kcal/mol)
1	-8.128
2	-8.113
3	-7.926
4	-7.872
5	-7.635
6	-7.529
7	-7.398
8	-7.380
9	-7.307
10	-7.300
11	-7.280
12	-7.254
13	-7.159
14	-7.111
15	-7.108
16	-7.040
17	-7.006
18	-6.905
19	-6.869
20	-6.844

Table S5. Docking energies and calculated binding affinity of compound **34** with AChE.

Model	Calculated affinity (kcal/mol)
1	-10.149
2	-10.034
3	-9.341
4	-9.164
5	-9.146
6	-9.076
7	-8.792
8	-8.495
9	-8.425
10	-8.406
11	-8.281
12	-7.926
13	-7.700
14	-7.241

Table S6. Docking energies and calculated binding affinity of compound **34** with BChE.

Model	Calculated affinity (kcal/mol)
1	-9.128
2	-9.021
3	-8.919
4	-8.720
5	-8.596
6	-8.465
7	-8.440
8	-8.386
9	-8.359
10	-8.100
11	-7.977
12	-7.944
13	-7.912
14	-7.897
15	-7.814
16	-7.796
17	-7.691
18	-7.645
19	-7.568
20	-7.315

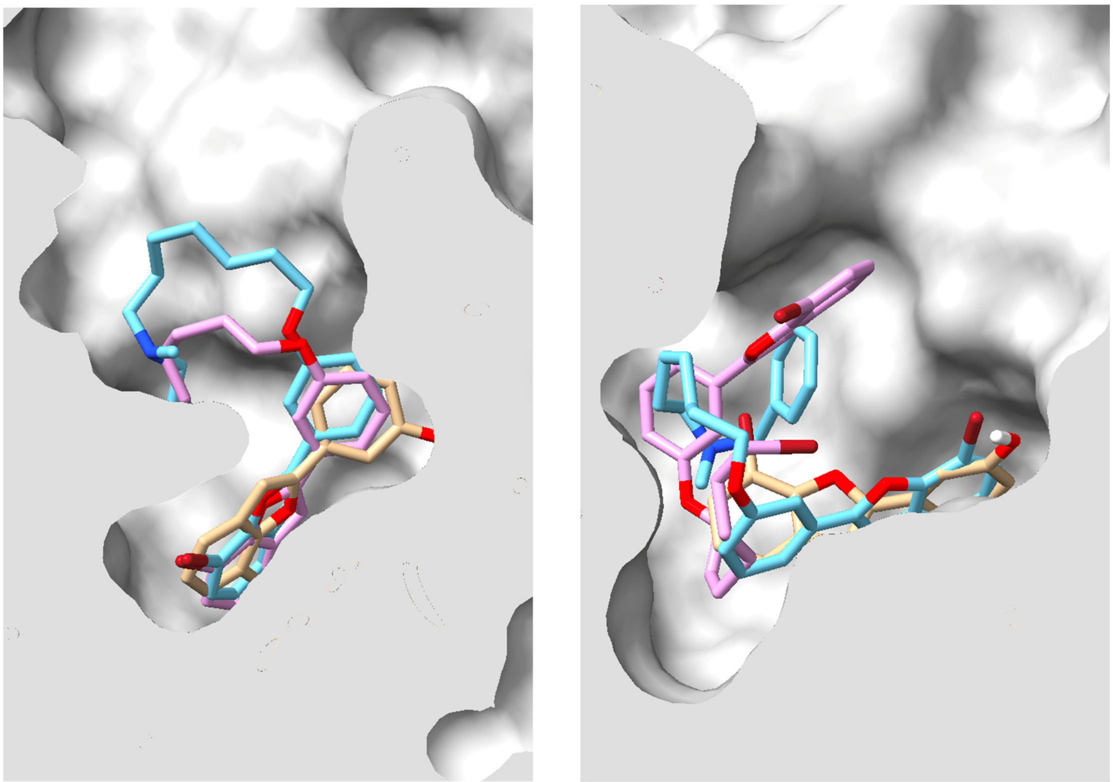

Figure S1. Superposition of compounds **34** (blue), **29** (pink) and **24** (yellow) inside AChE (left) and BChE (right) binding pockets. Only best scoring pose represented for each ligand.

5. References

[1] S. Rizzo, A. Tarozzi, M. Bartolini, G. Da Costa, A. Bisi, S. Gobbi, F. Belluti, A. Ligresti, M. Allarà, J-P. Monti, V. Andrisano, Vi. Di Marzo, P. Hrelia, A. Rampa. 2-Arylbenzofuran-based molecules as multipotent Alzheimer's disease modifying. European Journal of Medicinal Chemistry 2012, 58, 519-532; doi: 10.1016/j.ejmech.2012.10.067.

[2] G.L. Delogu, M.J. Matos, M. Fanti, B. Era, R. Medda, E. Pieroni, A. Fais, A. Kumar, F. Pintus. 2-Phenylbenzofuran derivatives as butyrylcholinesterase inhibitors: Synthesis, biological activity and molecular modeling. Bioorg Med Chem Lett. 2016, 26(9), 2308-13; doi: 10.1016/j.bmcl.2016.03.039.

[3] A. Kumar, F. Pintus, A. Di Petrillo, R. Medda, P. Caria, M.J. Matos, D. Viña, E. Pieroni, F. Delogu, B. Era, G.L. Delogu, A. Fais. Novel 2-pheynlbenzofuran derivatives as selective butyrylcholinesterase inhibitors for Alzheimer's disease. Sci Rep. 2018, 8(1), 4424; doi: 10.1038/s41598-018-22747-2.

[4] A. Fais, A. Kumar, R. Medda, F. Pintus, F. Delogu, M.J. Matos, B. Era, G.L. Delogu. Synthesis, molecular docking and cholinesterase inhibitory activity of hydroxylated 2-phenylbenzofuran derivatives. Bioorganic Chem. 2019, 84, 302-308; doi: 10.1016/j.bioorg.2019.05.026.

[5] G.L. Delogu, B. Era, S. Floris, R. Medda, V. Sogos, F. Pintus, G. Gatto, A. Kumar, G.T. Westermark, A. Fais. A new biological prospective for the 2-phenylbenzofurans as inhibitors of α-glucosidase and of the islet amyloid polypeptide formation. Int J Biol Macromol. 2021, 169, 428-35; doi: 10.1016/j.ijbiomac.2020.12.117.

[6] G.L. Delogu, A. Fais, F. Pintus, C. Goyal, M.J. Matos, B. Era, A. Kumar. Structural Insight of New Butyrylcholinesterase Inhibitors Based on Benzylbenzofuran Scaffold. Pharmaceuticals 2022, 15(3), 304. doi.org/10.3390/ph15030304.
